# Altered dorsal CA1 neuronal population coding in the APP/PS1 mouse model of Alzheimer’s disease

**DOI:** 10.1038/s41598-020-58038-y

**Published:** 2020-01-23

**Authors:** Udaysankar Chockanathan, Emily J. Warner, Loel Turpin, M. Kerry O’Banion, Krishnan Padmanabhan

**Affiliations:** 1 Department of Neuroscience, University of Rochester School of Medicine & Dentistry, Rochester, NY United States; 2 Neuroscience Graduate Program, University of Rochester School of Medicine & Dentistry, Rochester, NY United States; 3 Medical Scientist Training Program, University of Rochester School of Medicine & Dentistry, Rochester, NY United States; 4 Center for Visual Science, University of Rochester School of Medicine & Dentistry, Rochester, NY United States; 5 Ernest J. Del Monte Institute for Neuroscience, University of Rochester School of Medicine & Dentistry, Rochester, NY United States

**Keywords:** Computational neuroscience, Alzheimer's disease, Neural circuits

## Abstract

While the link between amyloid β (Aβ) accumulation and synaptic degradation in Alzheimer’s disease (AD) is known, the consequences of this pathology on population coding remain unknown. We found that the entropy, a measure of the diversity of network firing patterns, was lower in the dorsal CA1 region in the APP/PS1 mouse model of Aβ pathology, relative to controls, thereby reducing the population’s coding capacity. Our results reveal a network level signature of the deficits Aβ accumulation causes to the computations performed by neural circuits.

## Introduction

Alzheimer’s disease (AD) is a progressive neurodegenerative disorder associated with cognitive decline that is thought to arise in part from the pathological accumulation of amyloid β (Aβ) plaques^1^ throughout the neocortex and hippocampus. Plaques cause a constellation of changes in neural circuits including, but not limited to, degradation of dendritic spines^2^, reductions in synapse density^2,3^, and increases in the intrinsic excitability of neurons^3^. Aβ pathology has been linked to various behavioral and cognitive changes^4,5^; for example in mouse models, plaque burden correlates with degradation of place fields in the dorsal CA1 (dCA1) subfield of the hippocampus, and is associated with poor performance on spatial memory tasks^4^. Such behaviors require the orchestration of activity across large groups of neurons, or ensembles, whose dynamics are governed by the structure of neural circuits^6^. However, although Aβ pathology disrupts multiple features of these circuits^2,7,8^, the net effect of these changes on the structure of population activity and the resulting disruptions in neural computation remains unknown.

To address this question, we performed electrophysiological recordings in the hippocampus of awake APP/PS1 mice (model of Aβ pathology^9^), where amyloid plaques can be seen at 12 months of age^10^ and plaque burden corresponds to poor performance on spatial cognition and memory tasks, such as the T-maze alternation task^4,5^. We found a reduction in the pair-wise correlations between neurons in APP/PS1 animals as compared to controls. Additionally, we identified a reduction in the entropy of population activity across a large array of ensemble sizes, suggesting that the coding vocabulary of populations of neurons in the dCA1 region of hippocampus is compromised in this mouse model of amyloid pathology.

## Materials and Methods

### Animals

All protocols and procedures were approved by the University Committee on Animal Resources (UCAR) at the University of Rochester and were performed in accordance with the guidelines of the Institutional Animal Care and Use Committee (IACUC) at the University of Rochester. 4 APP/PS1 double transgenic mice and 4 littermate control mice were used in this study. The APP/PS1 mice expressed chimeric mouse/human amyloid precursor protein (Mo/HuApp695swe)^11^ and mutant human presenilin-1 (PS1-dE9)^12^ under the control of the neuron-specific prion protein promotor element^9^. All mice were males aged 11 to 13 months. By this age, amyloid plaques are present in dCA1^10,13^ and behavioral deficits manifest in multiple domains, including spatial memory, cognition, and anxiety^5,14–16^. This study was restricted to males, as it is known that there are sex differences in amyloid burden and cognitive changes in mouse models of Aβ pathology. For instance, female APP/PS1 mice show higher levels of amyloid plaques, as well as Aβ40 and Aβ42 peptides, than age-matched male mice^17^. Moreover, a recent study using the related Tg2576 mouse model, showed that the relationship between cognitive impairment and Aβ levels itself depends on sex, with female animals exhibiting poorer performance on a reference memory task than males with a similar amyloid burden^18^. Additionally, multiple aspects of hippocampal structure and function, including LTP induction^19^, seizure threshold^20^, dendritic morphology^21^, and synapse density^22^, fluctuate across the estrous cycle. Thus, we restricted our study to males. However, given the evidence for different prevalence of AD among human males and females^23,24^, studying sex-specific differences in neuronal population activity in the context of amyloid pathology remains a direction for future research beyond the scope of this study.

### Surgery

Prior to surgery, mice were anesthetized with an inhaled 1–2% isoflurane mixture. The scalp was resected and a 3D printed head frame made from polylactic acid (PLA) was affixed to the dorsal surface of the skull using veterinary adhesive (Vetbond, The 3 M Company, Maplewood, MN, USA) and dental cement (Ortho-Jet Powder and Jet Liquid, Lang Dental Mfg. Co., Wheeling, IL, USA). A metal screw was also implanted in the skull to serve as an electrical ground. For 72 hours, mice received postoperative 0.03 mL subcutaneous injections of 0.3 mg/mL buprenorphine every 12 hours.

### Run-wheel training

For 7 days following headframe implantation, mice were placed on a non-motorized running wheel in the recording rig for 1 hour daily. Mice were secured in place via clamps to the headframe, but they were free to run on the wheel. During these training sessions, no electrophysiological recordings were performed, although running behavior was recorded via a rotational encoder attached to the wheel. The purpose of these sessions was to accustom the mice to running on a wheel in the head-fixed setup.

### Electrophysiology

Following the training period, mice were anesthetized using 1–2% inhaled isoflurane and a craniotomy was performed over the right dorsal CA1 (dCA1) region using stereotactic coordinates (−2.5 mm caudal, 1.5 mm lateral of bregma)^25^. Mice were then transferred to the running wheel and allowed to recover from anesthesia. A 4-shank, 128-channel nanofabricated silicon array^26^ was vertically lowered into dCA1 (Fig. S1). While the mice were awake and behaving on the running wheel, extracellular voltage recordings were collected at 30 kHz in the 500–3500 Hz frequency band. One electrophysiological recording session was performed per animal. Simultaneously, the instantaneous running velocity of the mouse was also collected.

### Spike identification and sorting

Details on the number of spikes and units eliminated at each step are shown in Fig. S1. All data analysis was performed in MATLAB (The Mathworks, Natick, MA, USA). To eliminate background activity, the mean signal from all 128 channels was subtracted from the signal from each individual channel. Intervals during which the voltage signal surpassed a threshold of 8 standard deviations (SD) above or below 0 were tagged as putative action potentials, or spikes. Subsequently, a raster of all putative spikes was produced and intervals containing high-voltage artifacts that manifested on nearly all channels were visually identified and eliminated. Some putative spike waveforms crossed the 8 SD threshold at both positive and negative voltages; these double-counted putative spikes were identified and consolidated. Putative spikes that remained at the end of these pruning steps were preserved as true spikes.

**Figure 1:**
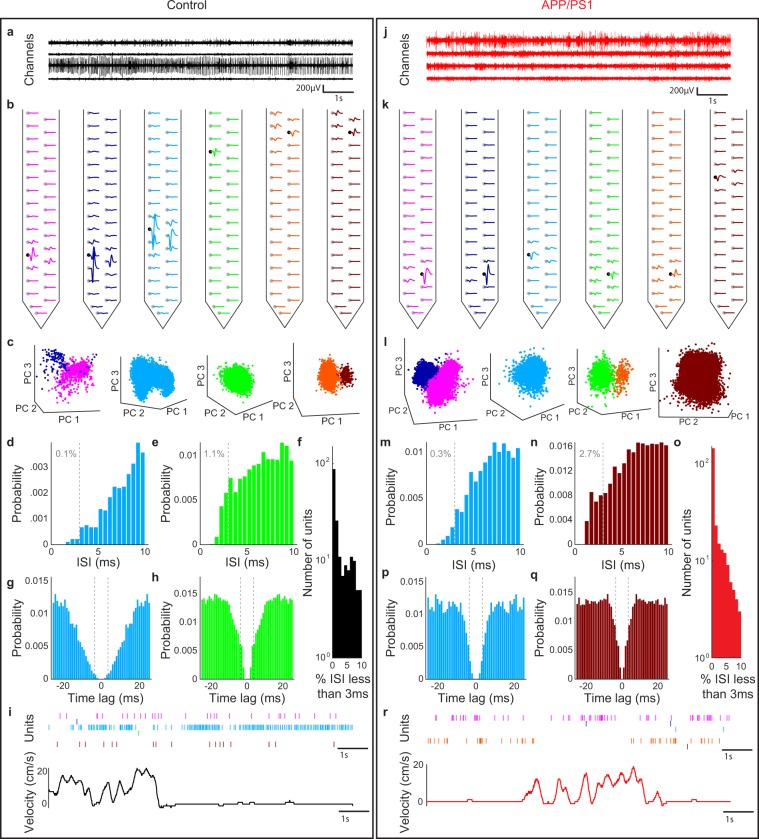
Isolation of single unit activity in a control (left) and APP/PS1 (right) animal from high-density arrays. (**a**,**j**) 10-second interval of raw electrophysiology data from four channels. (**b**,**k**) Mean waveform of each unit from channels in (**a**,**j**) as it appeared on the channel on which it was detected (black circle) and on the other channels of the electrode shank (grey circles). Colors of waveforms correspond to those in panels (**c**–**e**,**g**,**h**,**l**–**n**,**p**,**q**). (**c**,**l**) Projection of spike waveforms onto first three principal components of all waveforms from each channel. (**d**,**e**,**m**,**n**) Inter-spike interval (ISI) distributions for representative units. Percentage of ISI’s less than 3 ms (gray dashed line) is indicated in grey for control (**d,e**) and APP/PS1 (**m,n**) example units. (**f**,**o**) Proportion of ISI less than 3 ms for units in all (**f**) control and (**o**) APP/PS1 animals. (**g**,**h**,**p**,**q**) Spike count autocorrelations for representative units for (**g**,**h**) control and (**p**,**q**) APP/PS1 example units. Gray dashed line corresponded to +/−3 ms from the 0 lag autocorrelation time window. (**i**,**r**) Raster plot of sorted units and running velocity for a (**i**) control and (**r**) APP/PS1 animal. Each tick denotes an action potential.

Next, spikes were assigned to different neurons, or putative single units. The waveforms of all spikes from each channel were concatenated with those from 8 neighboring channels and projected onto a low-dimensional space using principal components analysis (PCA). The dimensionality of this space was defined by the smallest number of principal components that, when combined, would explain >80% of the variance in the waveforms. Spikes corresponding to different putative units appeared as distinct clusters. These were automatically identified using a mixture of Gaussians model^27^ (Fig. 1c,l).

The mean waveform of each putative unit across multiple channels was visualized (Fig. 1b,k) and manually evaluated by two reviewers. Putative units with noisy or symmetric waveforms, as well as those with fewer than 50 spikes, were discarded. Only the units that passed the evaluation of both reviewers were preserved for subsequent analysis. Additionally, groups of putative units with highly similar waveforms were merged. Pairs of units from different channels that had highly overlapping spike times were tagged as likely originating from the same neuron; one of these was discarded. Finally, an inter-spike interval distribution and autocorrelation was calculated for each unit (Figs. 1d–g,m–p, S2 and S3); those with over 10% of their inter-spike intervals less than 3 ms were discarded (Fig. 1h,q).

### Running behavior

The run-wheel was not motorized and its movement was controlled entirely by the mouse, which could run in either forward or reverse directions or remain stationary. Wheel movement was recorded by a 2-bit rotational encoder at 30 kHz. This was converted to units of cm/s by averaging the velocity across a 150 ms bin in time steps of 10 ms. Epochs of running were denoted as intervals when the run velocity exceeded 1 cm/s. All other times were denoted as stationary epochs.

### First and second order statistics

The mean firing rate (FR) of each unit was calculated by dividing the total number of spikes generated by that unit by the duration of the recording. To calculate pairwise correlations, instantaneous firing rate time-series were generated using a sliding window on the binary spike trains and converted to a z-score. The correlation coefficient was then calculated using these normalized time-series for every pair of units in each animal. Shuffled spike-trains were used to assess the significance of changes in correlation across running and stationary states within a single animal. For each unit, a shuffled spike-train with mean firing rate identical to that of the corresponding empirical spike-train was generated and converted to an instantaneous firing rate time-series. Pairwise correlations between the resulting time-series were calculated across stationary and running epochs. This shuffling process was repeated 1000 times for each animal.

### Entropy

The spike train of each unit was binarized over 10 ms non-overlapping windows. If that unit spiked at least once in that interval, it was denoted with a 1, and if it was silent throughout that interval, it was denoted with a 0. To estimate the entropy for each animal, 1000 random subsamples of 10 units were generated (Fig. S5). In each subsample, the state of all 10 units at a given time was denoted as a pattern (these patterns are also called words^28–30^). The relative frequency of the 2^10^ patterns in each subsample was computed and used to generate a pattern probability distribution. ${\sum }_{i=1}^{1024}-{p}_{i}lo{g}_{2}{p}_{i}$ was then used on the pattern probability distribution to calculate the entropy of each subsample. Entropy conditioned on running or stationary states was calculated by using spike trains taken only from running or stationary epochs, respectively.

Shuffled spike-trains were used to assess the significance of changes in entropy across running and stationary states within a single animal. For each unit, a shuffled spike-train with mean firing rate identical to that of the corresponding empirical spike-train was generated. Entropy conditioned on running or stationary states was then calculated for the resulting population of shuffled spike trains. This shuffling process was repeated 1000 times for each animal. To compare entropy differences at the single animal level, the average entropy from 250 samples in each animal was calculated and compared for the control animals versus the APP/PS1 animals. To ensure that the mean differences were not significant because of chance, we repeated this procedure 5000 times (bootstrap-without replacement).

### Maximum-entropy models

Maximum-entropy models^29,31^ were fit using the maxent_toolbox software^32^. First, the intrinsic spiking bias terms (**h**_i_) and pairwise interaction terms (**J**_ij_) were computed from the binned and binarized spike trains. The resulting model was then used to predict the probability of each pattern: ${\rm{P}}({{\rm{\sigma }}}_{1}{{\rm{\sigma }}}_{2}\ldots {{\rm{\sigma }}}_{{\rm{N}}})=\frac{1}{{\rm{Z}}}{{\rm{e}}}^{{\sum }_{{\rm{i}}}{{\rm{h}}}_{{\rm{i}}}{{\rm{\sigma }}}_{{\rm{i}}}}$ for the independent firing model and ${\rm{P}}({{\rm{\sigma }}}_{1}{{\rm{\sigma }}}_{2}\ldots {{\rm{\sigma }}}_{{\rm{N}}})=\frac{1}{{\rm{Z}}}{{\rm{e}}}^{{\sum }_{{\rm{i}}}{{\rm{h}}}_{{\rm{i}}}{{\rm{\sigma }}}_{{\rm{i}}}+\frac{1}{2}{\sum }_{{\rm{i}}\ne {\rm{j}}}{{\rm{\sigma }}}_{{\rm{i}}}{{\rm{\sigma }}}_{{\rm{j}}}}$ for the pairwise interactions model. σ_i_ denotes the binary state of each neuron and Z denotes the partition function, which normalizes the pattern probability distribution. The model pattern probabilities were then compared with the empirical pattern probabilities using the Kullback-Liebler divergence (KLD)^33^. The smaller the KLD, the better the agreement between the predicted and model probability distributions.

### Hypothesis testing

Statistical hypothesis testing was performed in R^34^ (The R Foundation for statistical computing, Vienna, Austria). To calculate effect sizes, the effsize package^35^ was used. See Table S1 for summary of results from hypothesis tests and confidence intervals. As the data were not normally distributed, hypothesis testing was performed using the two-sided Wilcoxon rank sum test.

## Results

To study the effect of amyloid pathology on network activity in awake animals, high-density 128 channel arrays were targeted to the dCA1 region in head-fixed APP/PS1 and littermate non-transgenic control mice (n = 4 male APP/PS1, n = 4 male control, age = 11–13 months) trained to run freely on a one-dimensional wheel. A representative example of band passed (500–3500 Hz) voltage traces for 4 of the 128 channels in a control (Fig. 1a) and an APP/PS1 mouse (Fig. 1j) reveal spiking activity from multiple neurons. From the raw traces, we identified spike waveforms for individual representative units on a single probe shank for a control (Fig. 1b) and APP/PS1 (Fig. 1k) by concatenating putative spikes across neighboring channels, projecting the concatenated waveforms onto a low-dimensional space using principal components analysis (PCA), and identifying clusters using a mixture of Gaussians model^27^ (Fig. 1c,l). This method allowed us to identify the activity from putative single units (Fig. S1**)**, whose waveforms could be detected across multiple neighboring channels. To better isolate the activity of individual units, we next examined the inter-spike interval distributions of each unit, with two representative examples shown for a control animal (Fig. 1d,e) and an APP/PS1 animal (Fig. 1m,n). Only units where fewer than 10% of the inter-spike intervals were less than 3 ms were included in our analysis (control = 165 units, APP/PS1 = 224 units, Figs. 1f,o, S2). Finally, we examined the temporal autocorrelation functions, shown for two representative examples in control and APP/PS1 animals (Figs. 1g,h,p,q, S3), and used this to identify single units, as has been done previously in dorsal CA1^36,37^. While we found up to 128 well isolated units in a single animal, on average we identified 56 ± 48 units in the control animals and 41 ± 17 units in the APP/PS1 animals. When we examined the spike waveforms from the units isolated in the control and the APP/PS1 animals, we found no significant differences (Fig. S4), suggesting that there was no bias indicative of different types of neurons being sampled between control and APP/PS1 mice. Finally, the spiking activity from these single units was then mapped back onto the behavior we studied, running, for the control (Fig. 1i) and APP/PS1 mice (Fig. 1r).

**Figure 2:**
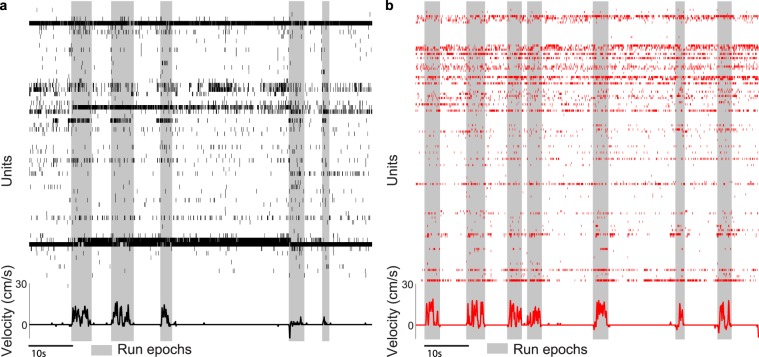
High-density awake recordings of dCA1 neuronal populations reveal complexity of activity patterns. (**a**,**b**) Raster plot of all units isolated in a representative control (**a**) and APP/PS1 (**b**) animal and simultaneous running behavior.

**Figure 3:**
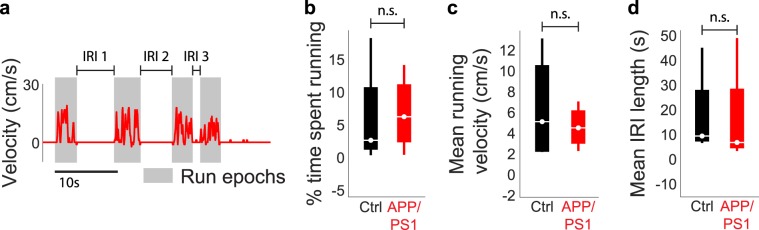
Statistics of running behavior are similar between control and APP/PS1 mice. (**a**) Example run velocity trace. Run epochs are shaded in grey and three inter-run intervals (IRI) are labeled. (**b**–**d**) No significant differences in (**b**) proportion of time spent running, (**c**) mean running velocity, or (**d**) mean IRI length between the control and APP/PS1 groups. For all box plots, box center denotes the median, box edges denote the interquartile range (IQR), upper whisker extends to the largest value smaller than 1.5 × IQR from upper edge of box, and lower whisker extends to the smallest value larger than 1.5 × IQR from lower edge of box.

Population rasters of single unit activity from representative ensembles of neurons in a control (Fig. 2a) and an APP/PS1 mouse (Fig. 2b) illustrated the temporal complexity of activity within a single animal during periods when the animal was either stationary or running (shaded in grey). When we examined the statistics of running behavior (Fig. 3a), including proportion of time spent running (mean ± std: control = 6.05% ± 8.28%, APP/PS1 = 6.84% ± 5.87%, *p* = 0.69, two-sided Wilcoxon rank-sum test, n = 4 control mice, 4 APP/PS1 mice, Figs. 3b, S6a), running velocity (mean ± std: control = 6.42 ± 5.27 cm/s, APP/PS1 = 4.63 ± 2.07 cm/s, *p* > 0.99, two-sided Wilcoxon rank-sum test, n = 4 control mice, 4 APP/PS1 mice, Figs. 3c, S6b), and duration of inter-run intervals (mean ± std: control = 17.8 ± 18.4 s, APP/PS1 = 16.6 ± 21.8 s, *p* = 0.69, two-sided Wilcoxon rank-sum test, n = 4 control mice, 4 APP/PS1 mice, Figs. 3d, S6c), we found no significant differences between control and APP/PS1 mice, consistent with previous behavioral studies that have shown no deficits in motor function in APP/PS1 mice relative to non-transgenic controls^5^.

While a number of previous studies have examined properties of neuronal activity in anesthetized control and APP/PS1 animals in the CA1 regions of hippocampus^3,7,38,39^, other groups have shown that anesthesia substantially alters the statistics of neural activity^40,41^. Thus, less is known about neural activity in awake APP/PS1 animals, particularly at the population level. To address this, we first calculated mean firing rates of neurons in each group and observed that those in APP/PS1 mice had lower firing rates than those of control mice (mean ± std: control = 1.65 ± 3.32 Hz, APP/PS1 = 0.80 ± 1.49 Hz, *p* < 10^−3^, two-sided Wilcoxon rank-sum test, n = 165 units from 4 control mice, 224 units from 4 APP/PS1 mice, Fig. S7). Although the mean firing rates were lower in the APP/PS1 animals, we also observed examples of high firing rate neurons, suggesting the presence of the hyperactive and hypoactive subpopulations of neurons that other groups have identified in mouse models of AD^3,7,38^.

**Figure 4:**
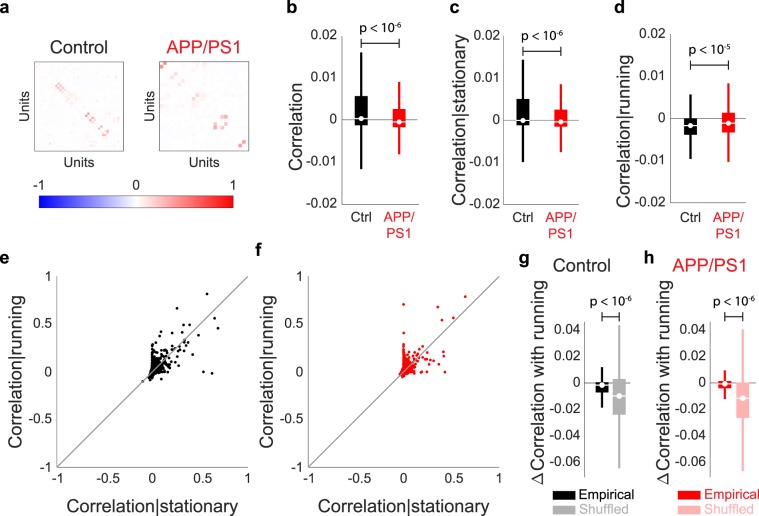
Spike count correlations of dCA1 neuronal populations are different between control and APP/PS1 animals. (**a**) Matrix of correlation coefficients for a representative control (left) and APP/PS1 (right) mouse. (**b**) Mean overall correlations, as well as correlations during (**c**) stationary epochs were lower in APP/PS1 mice as compared to control mice. (**d**) Correlations during running epochs were increased in APP/PS1 mice as compared to control mice. (**e**,**f**) Scatter plots showing change in correlations between stationary and running epochs for each pair of units in (**e**) control and (**f**) APP/PS1 mice. (**g**,**h**) Changes in correlation with running are significantly different than shuffled distributions for (**g**) control and (**h**) APP/PS1 mice. For all box plots, box center denotes the median, box edges denote the interquartile range (IQR), upper whisker extends to the largest value smaller than 1.5 × IQR from upper edge of box, and lower whisker extends to the smallest value larger than 1.5 × IQR from lower edge of box.

Next, to investigate how the activity of individual neurons were organized and related to each other, we first quantified the pair-wise correlation between neurons in both the control and APP/PS1 animals (Fig. 4a). Correlations have been tied to behaviors ranging from spatial navigation^42,43^ to attention^44,45^, and alterations in correlations may be an early marker of neurologic disorders^46^. We found that correlations were decreased in the APP/PS1 mice, relative to controls (median ± std: control = 4.05 × 10^−4^ ± 3.68 × 10^−2^, APP/PS1 = −3.95 × 10^−4^ ± 1.84 × 10^−2^, *p* < 10^−6^, two-sided Wilcoxon rank-sum test, n = 3733 unit-pairs from 4 control mice, 9653 unit-pairs from 4 APP/PS1 mice, Figs. 4b, S8). Additionally, we found that differences in correlation were significant when the animals remained stationary (median ± std: control = −3.71 × 10^−5^ ± 3.72 × 10^−2^, APP/PS1 = −3.75 × 10^−4^ ± 1.84 × 10^−2^, *p* < 10^−6^, two-sided Wilcoxon rank-sum test, n = 3733 unit-pairs from 4 control mice, 9653 unit-pairs from 4 APP/PS1 mice, Fig. 4c) or when they ran (median ± std: control = −1.69 × 10^−3^ ± 7.66 × 10^−2^, APP/PS1 = −1.12 × 10^−3^ ± 2.55 × 10^−2^, *p* < 10^−5^, two-sided Wilcoxon rank-sum test, n = 3733 unit-pairs from 4 control mice, 9653 unit-pairs from 4 APP/PS1 mice, Fig. 4d), suggesting that the interactions between neurons were influenced by the behavior of the animal. Finally, we determined how correlations changed within animals *across* the stationary and running epochs (Fig. 4e,f). In both control (median ± std: empirical = −1.61 × 10^−3^ ± 7.18 × 10^−2^, shuffled = −9.84 × 10^−3^ ± 6.11 × 10^−2^, *p* < 10^−6^, two-sided Wilcoxon rank-sum test, n = 3733 unit-pairs from 4 control mice, Fig. 4g) and APP/PS1 animals (median ± std: empirical = −6.57 × 10^−4^ ± 2.14 × 10^−2^, shuffled = −1.15 × 10^−2^ ± 1.96 × 10^−1^, *p* < 10^−6^, two-sided Wilcoxon rank-sum test, n = 9653 unit-pairs from 4 APP/PS1 mice, Fig. 4h), we found a significant change in correlations with running as compared to shuffled data, further suggesting that running behavior exerts a significant effect on the interactions between neurons across populations.

**Figure 5:**
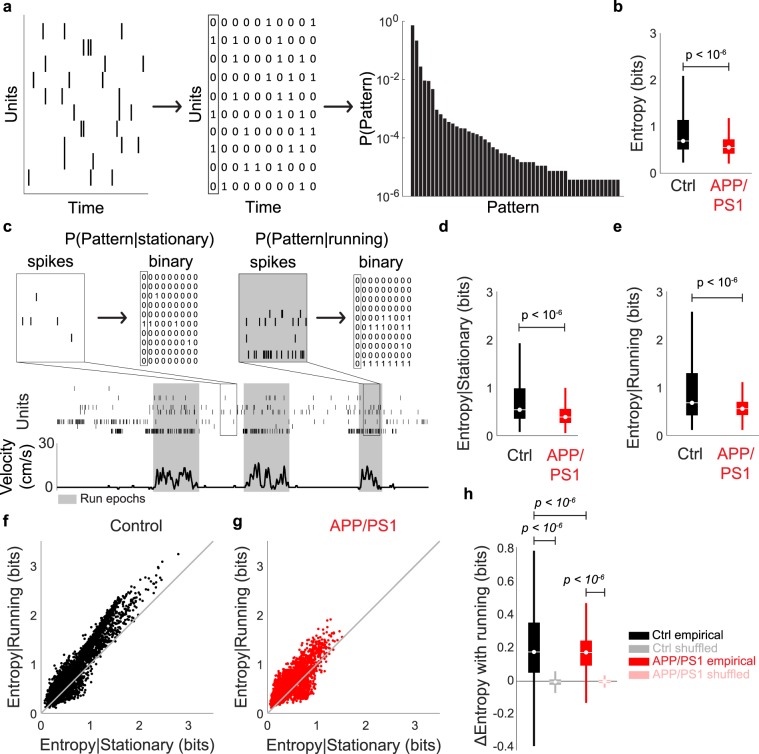
Entropy of dCA1 neuronal populations is different between control and APP/PS1 animals. (**a**) Schematic of entropy calculation. (**b**) APP/PS1 mice had significantly lower entropy than control mice. (**c**) Schematic of entropy calculation conditioned on behavioral state. (**d**,**e**) For both (**d**) stationary and (**e**) running conditions, the entropy of APP/PS1 animals was lower than that of controls. (**f**,**g**) Scatter plot comparing entropy when the animal is stationary versus running for (**f**) control and (**g**) APP/PS1 mice. Each point represents a single population of neurons. (**h**) Entropy change with running. Both control and APP/PS1 animals exhibited a significant increase in entropy with running, relative to shuffled spike trains. However, the magnitude of this increase was larger in controls than APP/PS1 animals. For all box plots, box center denotes the median, box edges denote the interquartile range (IQR), upper whisker extends to the largest value smaller than 1.5 × IQR from upper edge of box, and lower whisker extends to the smallest value larger than 1.5 × IQR from lower edge of box.

While correlations provided a glimpse into network disruption in APP/PS1 mice, they only measure interactions between pairs of neurons. To further investigate how collective changes in activity across the entire population were altered in the APP/PS1 animals, we calculated an alternative measure, entropy, that quantifies the diversity of firing patterns across ensembles of neurons over the duration of the recording^31,47^ (Figs. 5a, S5 and S9). First, we observed that the entropy of dCA1 populations in APP/PS1 animals was decreased relative to that of controls (mean ± std: control = 0.70 ± 0.42 bits, APP/PS1 = 0.45 ± 0.24 bits, *p* < 10^−6^, two-sided Wilcoxon rank-sum test, n = 4000 points (1000/animal) from 4 control mice and 4000 points (1000/animal) from 4 APP/PS1 mice, Fig. 5b), suggesting that the diversity of patterns of activity generated by equivalently sized ensembles of neurons in APP/PS1 animals was smaller than in controls. The reduced entropy in APP/PS1 animals was significant across a range of ensemble sizes varying from 3 to 19 neurons and across multiple timescales of spike binning (Fig. S10). Furthermore, the reduction in entropy between APP/PS1 and control animals was significant even after correcting for the different firing rates between the two groups (Fig. S11) and significant when the data were analyzed at the single-animal level (mean ± std: control = 0.70 ± 0.30 bits, APP/PS1 = 0.45 ± 0.16 bits, *p* = 0.03, two-sided Wilcoxon rank-sum test, n = 4 control mice, 4 APP/PS1 mice, Fig. S12).

Since the entropy in both the control and the APP/PS1 animals was calculated over the entire recording duration, it included both running and stationary epochs. However, different behavioral states impacted properties of neuronal activity, such as correlations (Fig. 4)^43,48,49^. Thus, we wished to determine if the entropy was also altered by behavior, and the extent to which these alterations were different in control and APP/PS1 animals (Fig. 5c). First, running was associated with an increase in entropy in both control (mean ± std: stationary = 0.69 ± 0.42 bits, running = 0.89 ± 0.57 bits, *p* < 10^−6^, two-sided Wilcoxon rank-sum test, n = 4000 points (1000/animal) from 4 control mice, Figs. 5d–f,h, S13) and APP/PS1 mice (mean ± std: stationary = 0.44 ± 0.23 bits, running = 0.60 ± 0.25 bits, *p* < 10^−6^, two-sided Wilcoxon rank-sum test, n = 4000 points (1000/animal) from 4 APP/PS1 mice, Figs. 5d,e,g,h, S13). Interestingly, independent of whether the animal was stationary (mean ± std: control = 0.69 ± 0.42 bits, APP/PS1 = 0.44 ± 0.23, *p* < 10^−6^, two-sided Wilcoxon rank-sum test, n = 4000 points (1000/animal) from 4 control mice and 4000 points (1000/animal) from 4 APP/PS1 mice) or running (mean ± std: control = 0.89 ± 0.57 bits, APP/PS1 = 0.60 ± 0.25 bits, *p* < 10^−6^, two-sided Wilcoxon rank-sum test, n = 4000 points (1000/animal) from 4 control mice and 4000 points (1000/animal) from 4 APP/PS1 mice), the entropy was always lower in APP/PS1 animals as compared to the controls (Fig. 5d,e), suggesting that the reduction in the number of possible network patterns was a general feature of ensemble activity. Furthermore, the magnitude of the change in entropy from stationary to running was significantly smaller in APP/PS1 mice than controls (mean ± std: control = 0.20 ± 0.21 bits, APP/PS1 = 0.16 ± 0.16 bits, *p < *10^−6^, two-sided Wilcoxon rank-sum test, n = 4000 points (1000/animal) from 4 control mice and 4000 points (1000/animal) from 4 APP/PS1 mice, Fig. 5h). Thus, APP/PS1 animals had reductions not only in the number of patterns that could be generated by neural ensembles, but also in the flexibility of those patterns across behaviors. As no major differences in running (Fig. 3) were found between APP/PS1 mice and controls, it is unlikely that the changes in entropy were due to sampling population activity across different behaviors in the control and APP/PS1 animals.

While the changes in entropy observed in APP/PS1 animals suggested that the diversity of network patterns was reduced in transgenic animals, it remained unclear why such a reduction occurred. For instance, hypersynchronous neuronal activity, associated with the increased risk of seizures in human AD as well as mouse models^50,51^, could reduce entropy by increasing the occurrence of patterns of highly correlated neurons. By contrast, a similar, albeit mechanistically distinct reduction in entropy could occur due to the synapse loss and compromised dendritic structure seen in APP/PS1 animals^3^, which would also also result in fewer network patterns.

**Figure 6:**
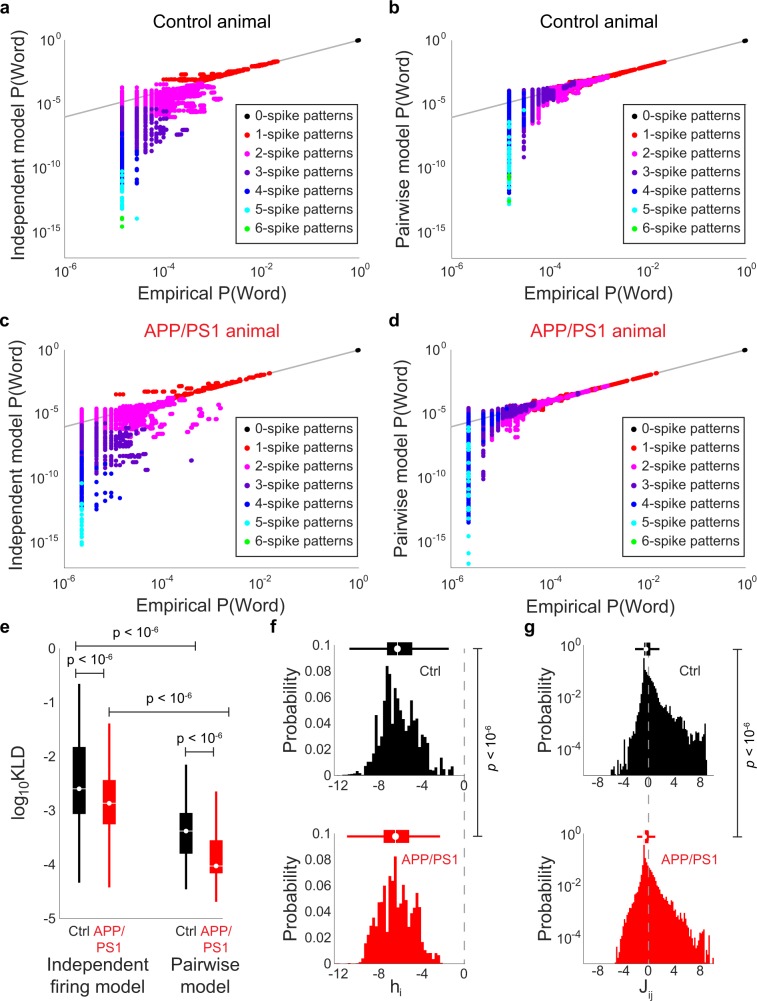
Maximum entropy models differentially predict dCA1 neuronal population activity in control and APP/PS1 mice (**a**-**d**) Scatter plot comparing predicted and model pattern probabilities for a representative (**a**,**b**) control and (**c**,**d**) APP/PS1 animal for the independent model (**a**,**c**) and pairwise model (**b**,**d**). Colors denote the number of coactive neurons in each pattern. (**e**) Kullback-Liebler divergence (KLD) between empirical and model pattern probability distributions. For both models, the KLD was smaller for APP/PS1 mice than controls. Moreover, the pairwise models for both control and APP/PS1 groups had a lower KLD than the corresponding independent models. (**f**,**g**) Histograms of (**f**) h_i_ and (**g**) J_ij_ terms for control (top) and APP/PS1 (bottom) animals. Dashed line denotes 0. Both h_i_ and J_ij_ terms were smaller in the APP/PS1 group than the controls. For all box plots, box center denotes the median, box edges denote the interquartile range (IQR), upper whisker extends to the largest value smaller than 1.5 × IQR from upper edge of box, and lower whisker extends to the smallest value larger than 1.5 × IQR from lower edge of box.

To disambiguate these different possibilities, we linked the statistics of network patterns to the functional coupling of neurons using maximum-entropy models that predict patterns of activity with as few *a priori* assumptions of structure as possible^29,31,43^. For each ensemble, we fit both an independent firing model that only contained a term for the activity of each neuron (**h**_i_) and a pairwise interaction model that contained not only the **h**_i_ term, but also a term for the functional coupling between pairs of neurons (**J**_ij_). This allowed us to estimate how such properties as intrinsic excitability and pairwise interactions collectively shaped the patterns of activity generated by neuronal ensembles and, in turn, the entropy. To visualize this, we first plotted the predicted frequency for each firing pattern from the maximum entropy model against the corresponding empirical frequency for a representative control (Fig. 6a,b) and APP/PS1 (Fig. 6c,d) animal. Each point represents a different pattern and the color denotes the number of active units in that pattern. To quantify the goodness of fits between model and data, we used a measure of the distance between two probability distributions, the Kullbeck-Liebler Divergence (KLD). First, the KLD of the independent firing model was significantly larger for controls than APP/PS1 animals (mean ± std: control = 1.76 × 10^−2^ ± 3.24 × 10^−2^, APP/PS1 = 3.91 × 10^−3^ ± 6.26 × 10^−3^, *p < *10^−6^, two-sided Wilcoxon rank-sum test, n = 4000 points (1000/animal) from 4 control mice and 4000 points (1000/animal) from 4 APP/PS1 mice, Figs. 6e, S14 and S15), showing that a first order maximum-entropy model better predicted patterns of neuronal activity for APP/PS1 animals than for controls. However, this difference was no longer significant if we corrected for differences in the mean firing rate between control and APP/PS1 mice (Fig. S11), not surprising as first order maximum entropy models are sensitive to firing rate changes. Furthermore, we found that the pairwise interactions model was also better at predicting patterns in APP/PS1 animals as compared to control animals (mean ± std: control = 7.65 × 10^−4^ ± 9.46 × 10^−4^, APP/PS1 = 2.26 × 10^−4^ ± 2.88 × 10^−4^, *p < *10^−6^, two-sided Wilcoxon rank-sum test, n = 4000 points (1000/animal) from 4 control mice and 4000 points (1000/animal) from 4 APP/PS1 mice, Figs. 6e, S14 and S16). Interestingly, this improved prediction of the pairwise model for APP/PS1 animals was preserved even after controlling for the mean firing rate differences between the two groups (Fig. S11), suggesting that one of the features of circuit level deficits in the APP/PS1 was the alteration in the interactions between neurons. To explore this link further, we examined the differences in the parameters of the maximum entropy models for both the excitability (**h**_i_ terms) and interactions (**J**_ij_ terms) in the control and the APP/PS1 animals. The **h**_i_ terms for the APP/PS1 animals were smaller than those for the controls (mean ± std: control = −5.96 ± 1.65, APP/PS1 = −6.26 ± 1.56, *p < *10^−6^, two-sided Wilcoxon rank-sum test, n = 4000 points (1000/animal) from 4 control mice and 4000 points (1000/animal) from 4 APP/PS1 mice, Figs. 6f, S17), which is consistent with the finding of decreased mean firing rates in the APP/PS1 animals (Fig. S7). Importantly, we also found that the **J**_ij_ values in the APP/PS1 animals were decreased as compared to the control animals (mean ± std: control = −0.02 ± 1.15, APP/PS1 = −0.25 ± 1.04, *p < *10^−6^, two-sided Wilcoxon rank-sum test, n = 4000 points (1000/animal) from 4 control mice and 4000 points (1000/animal) from 4 APP/PS1 mice, Figs. 6g, S18), similar to the decreased pairwise correlations we found in the APP/PS1 animals (Figs. 4b, S19).

The differences in the **h**_i_ and **J**_ij_ terms between APP/PS1 and control mice imply that both the single neuron activity and functional coupling between pairs of neurons are disrupted in APP/PS1 mice. To link these observations to the collective activity of larger ensembles of neurons, we examined how the maximum entropy models predicted the frequency of different network patterns grouped by the number of coactive neurons in those patterns. First, patterns with a high number of coactive neurons occurred less frequently in the APP/PS1 animals as compared to the controls (Fig. S20). The patterns of 6 or 7 coactive neurons in a 10 neuron population that occurred in the control group were all but absent in the APP/PS1 group.

In the pair-wise maximum entropy model, the occurrence of these large coactive groups of neurons was underestimated in the control as compared to the APP/PS1 animals, partially explaining the difference in the prediction error of the models between the two groups (Fig. S21). In the control animals, even including the pair-wise interactions was insufficient to estimate the occurrence of these highly coactive ensembles. By contrast, as such patterns were largely absent in the APP/PS1 animals, the pair-wise model with the diminished **J**_ij_ terms, was more successful at predicting the paucity of dCA1 representations observed in APP/PS1 animals.

## Discussion

In summary, the decreased entropy observed in APP/PS1 animals revealed a reduction in the diversity of network patterns available to populations of neurons in dCA1. Such a decrease constrains the number of patterns the network can generate, and could therefore limit the ability of dCA1 to encode sensory stimuli or store diverse experiences. Interestingly, it has been found that both theta oscillations and sharp waves/ripples are slower^52^ and that theta power is reduced^53^ in APP/PS1 mice. As this field activity is disrupted, the synchronization of spike times across ensembles of neurons may also be affected, the effect of which would be reducing the total entropy and compromising representations in the hippocampus.

Consistent with this idea, previous work has shown that place fields are much broader and carry less spatial information in a related mouse model of AD, the Tg2576 strain^4^. Interestingly, recent work where head-fixed mice were trained to run on a wheel similar to the one used in our experiments showed that both place and non-place cells are important for predicting the global state of the dCA1 network^43^. Understanding the statistics of network activity therefore likely requires accounting for both external variables (such as place) and what the authors referred to as “internal states”^43^. In this regard, the reduction in coding vocabulary observed in the APP/PS1 animals may reflect not only the spatial memory impairments seen in APP/PS1 mice^5^ and human AD^54^, but also the underlying cognitive^55,56^ and mood^57,58^ impairments that diminish function more broadly in AD.

Although there was a significant reduction in entropy in the APP/PS1 mice, we found that the pairwise maximum-entropy model was better at predicting dCA1 population activity in APP/PS1 animals as compared to control animals. Moreover, unlike the independent model, this improved prediction of pattern probabilities in the pair-wise model for APP/PS1 animals was still present even after controlling for mean firing rate differences between the two groups. This is particularly important for patterns with many coactive neurons, which are systematically underestimated in the control animals by the maximum entropy models; these same patterns were largely absent in the APP/PS1 animals. Additionally, as the pairwise model learns the reduced functional coupling in APP/PS1 animals, these weakened network interactions serve to better estimate the frequency of occurrence of patterns of activity.

While the parameters of the models do not explicitly map onto circuit features, such as synaptic connectivity or intrinsic excitability, they do provide insight into how the constellation of cellular and molecular changes in APP/PS1 and related models of AD may result in diminished coding capacity and network function^2,3^. Taken together, the pairwise model has more information to describe the diversity of network patterns in APP/PS1 animals as compared to the controls. By contrast, in control animals, the pairwise interactions learned by the model were insufficient to predict the diversity of observed network patterns. In the control animals, a number of instances occurred when 3 or more neurons were simultaneously active, and the pairwise model consistently underestimated the frequency of these occurrences. The higher-than expected frequency of occurrence of this synchronous network states suggests that the structure of activity patterns in control animals is likely shaped by higher order interactions^59^ (triplet, quadruplet, etc.) that are either diminished or absent in APP/PS1 animals. Previous studies have identified the role that these higher-order interactions play in marshalling population activity in sensory systems and implicated the local circuits that may give rise to such higher-order interactions^31^. Our results highlight the potential importance these high order interactions may have in orchestrating dCA1 activity and suggest that they may be especially vulnerable to the Aβ pathology in APP/PS1 animals.

## Supplementary information

Supplemental Figures.

## Data Availability

The datasets generated during and/or analyzed during the current study, including the raw data used to generate all 6 main figures and all supplemental figures, are available from the corresponding author on request.

## Code availability

The computer code used to generate the results of the current study are available from the corresponding author on request.
