# Supplementary material for: Altered dorsal CA1 neuronal population coding in the APP/PS1 mouse model of Alzheimer’s disease
Source: Sci Rep. 2020 Jan 23;10:1077. doi: 10.1038/s41598-020-58038-y (PMC6978514; doi:10.1038/s41598-020-58038-y)
Supplement: Supplementary file 1 — Supplemental Figures. [file 41598_2020_58038_MOESM1_ESM.pdf]

Altered dorsal CA1 neuronal population coding in the APP/PS1 mouse model of Alzheimer's disease

Udaysankar Chockanathan[1,2,3], Emily J. Warner[1,2], Loel Turpin[1], M. Kerry O'Banion[1,2,3,5], Krishnan Padmanabhan[1,2,4,5]

[1]Department of Neuroscience, University of Rochester School of Medicine & Dentistry

[2]Neuroscience Graduate Program, University of Rochester School of Medicine & Dentistry

[3]Medical Scientist Training Program, University of Rochester School of Medicine & Dentistry

[4]Center for Visual Science, University of Rochester School of Medicine & Dentistry

[5]Ernest J. Del Monte Institute for Neuroscience, University of Rochester School of Medicine & Dentistry

correspondence: Krishnan_padmanabhan@urmc.rochester.edu

Supplemental materials

Supplemental Figures: 21

Supplemental Tables: 1

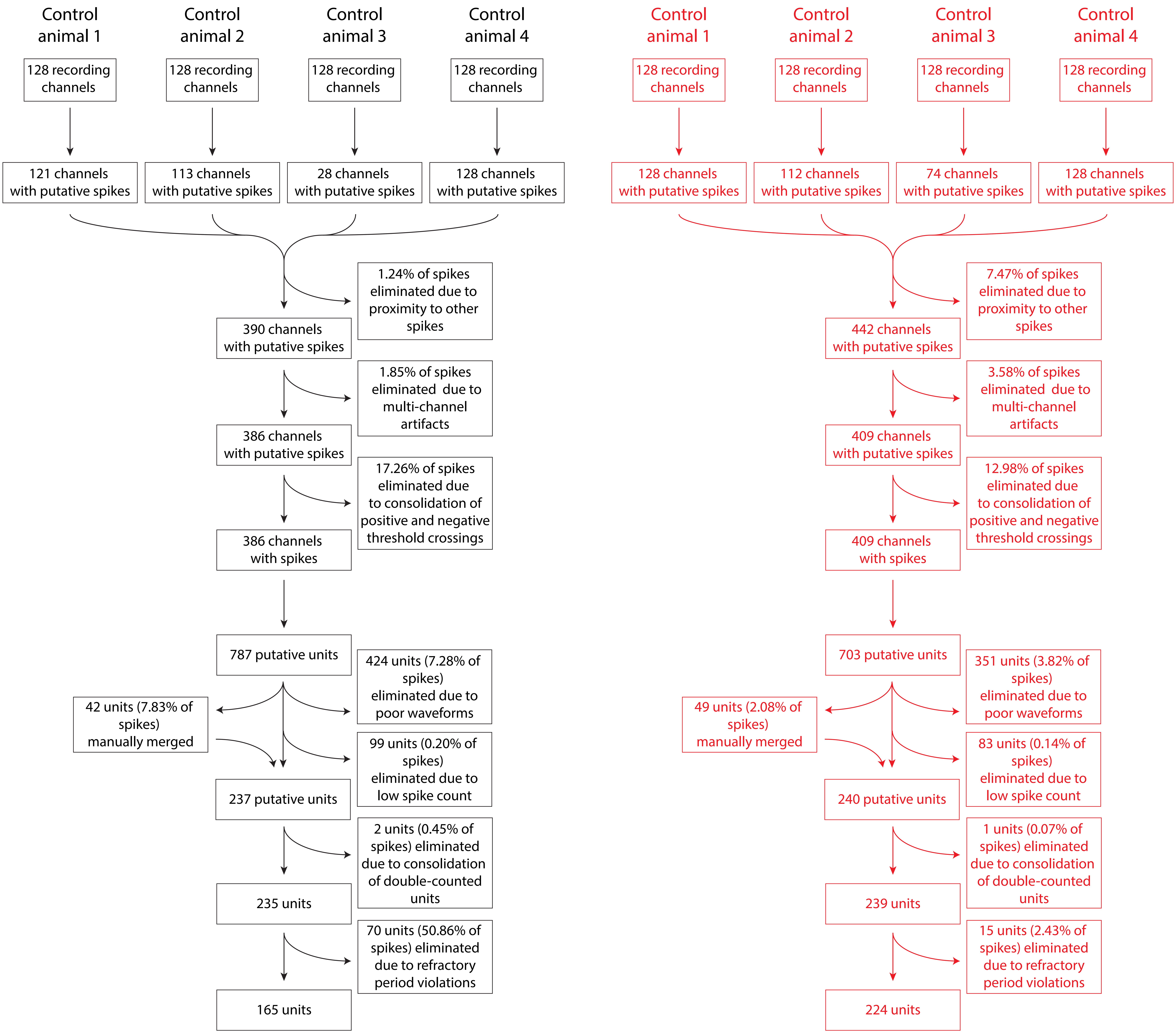

Figure S1 Schematic of spike sorting pipeline, indicating the number and proportion of spikes eliminated at each step.

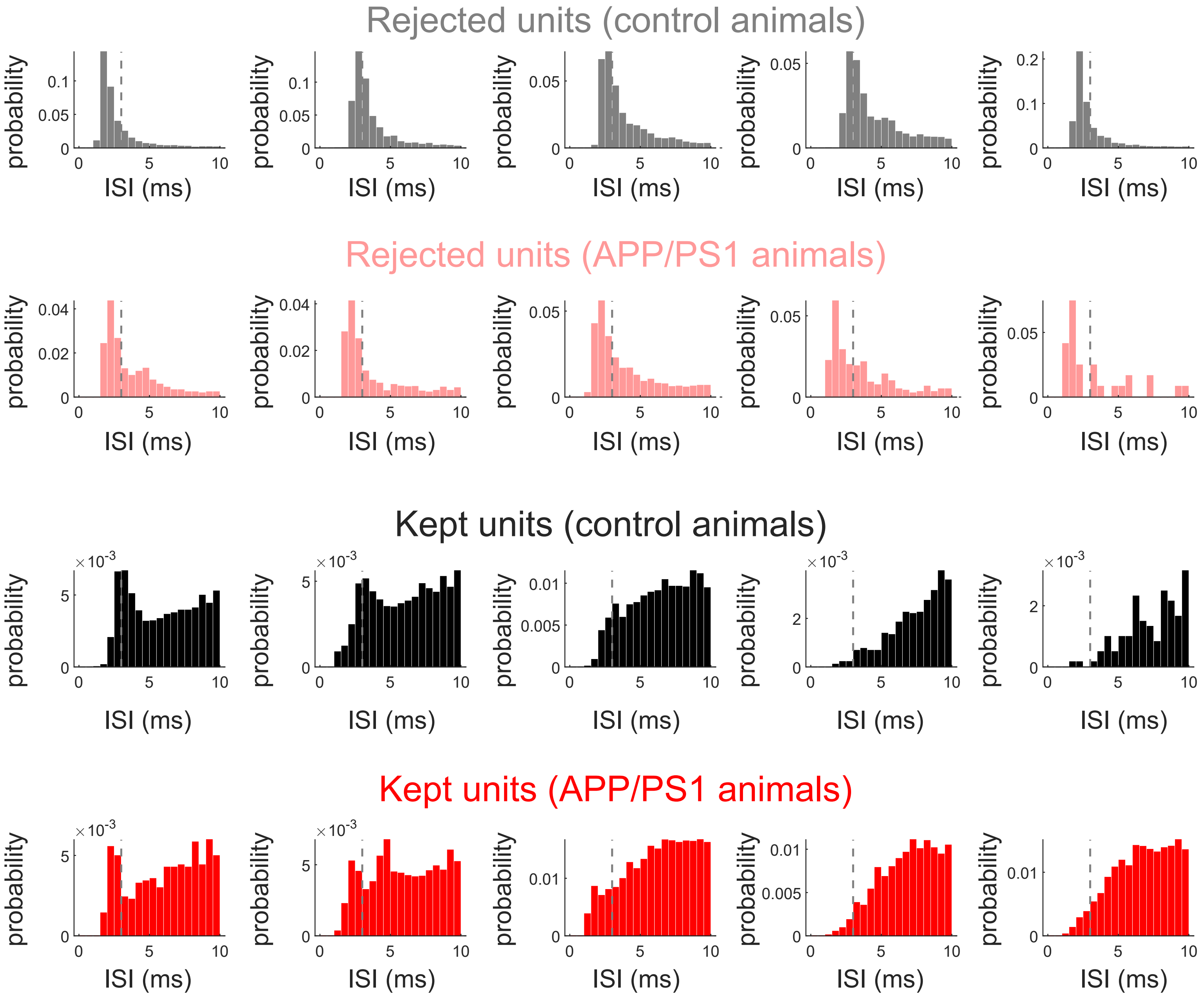

Figure S2 Representative examples of inter-spike interval distributions of (**a**,**b**) rejected and (**c**,**d**) kept units from control (**a**,**c**) and APP/PS1 (**b**,**d**) animals.

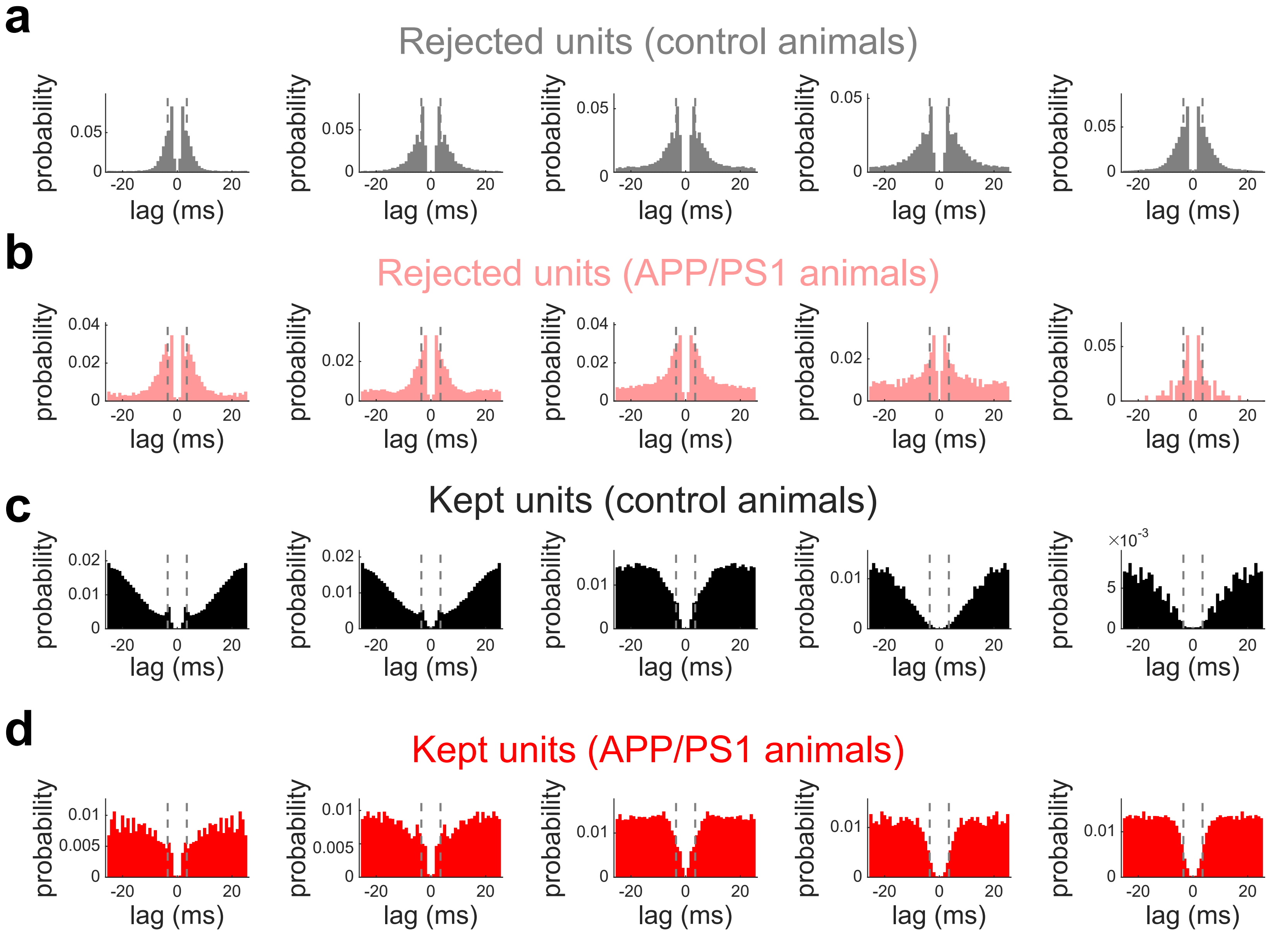

Figure S3 Representative examples of spike autocorrelations of (**a**,**b**) rejected and (**c**,**d**) kept units from control (**a**,**c**) and APP/PS1 (**b**,**d**) animals.

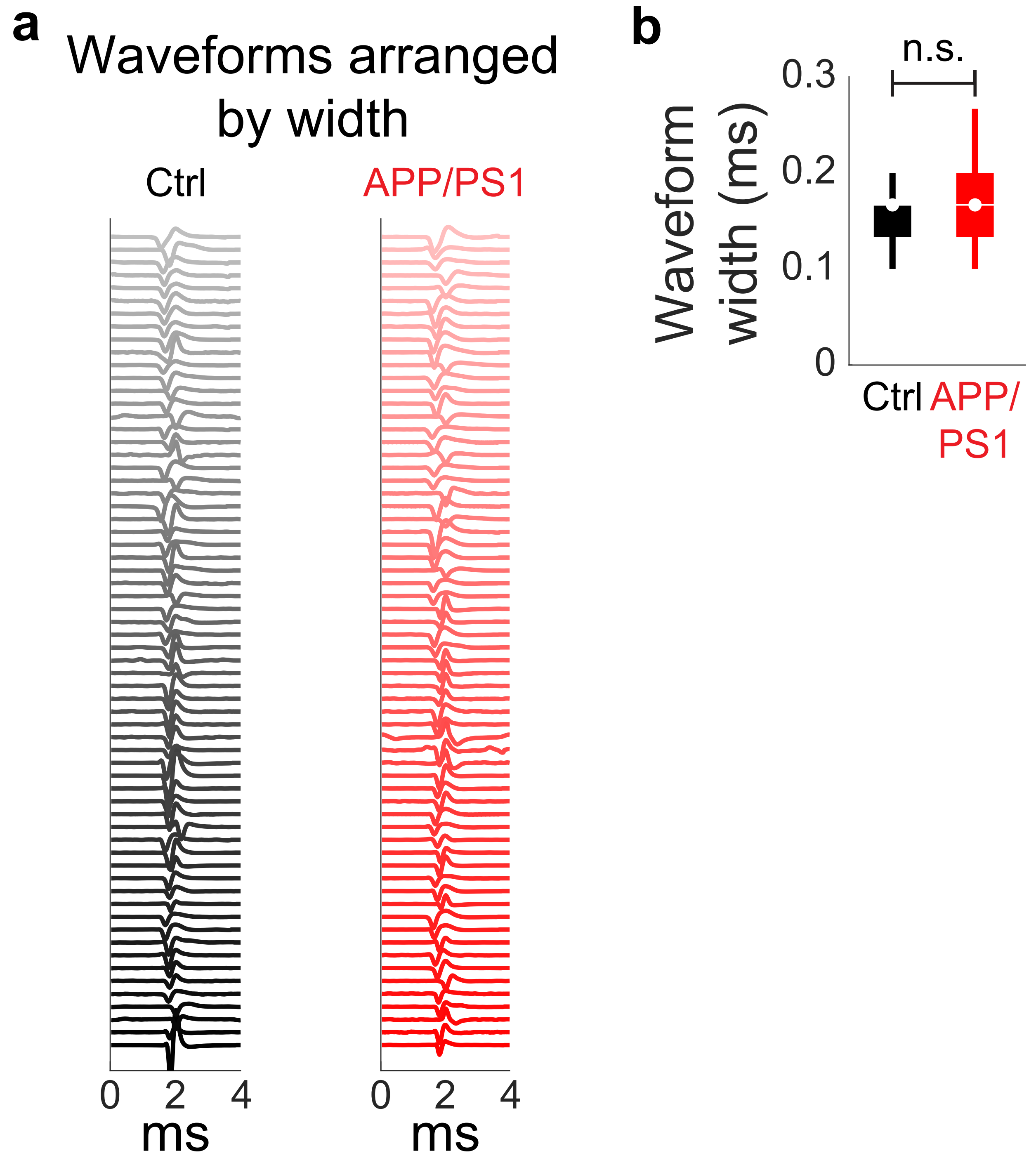

Figure S4 Characterization of waveform widths. (**a**) Mean unit waveforms from control (left) and APP/PS1 (right), arranged from bottom to top by increasing waveform width. (**b**) Box plot of waveform widths for control and APP/PS1 mice. There is no significant difference in the widths between the two groups (two-sided Wilcoxon rank sum test). Box plot center denotes the median, box edges denote the interquartile range (IQR), upper whisker extends to the largest value smaller than 1.5×IQR from upper edge of box, and lower whisker extends to the smallest value larger than 1.5×IQR from lower edge of box.

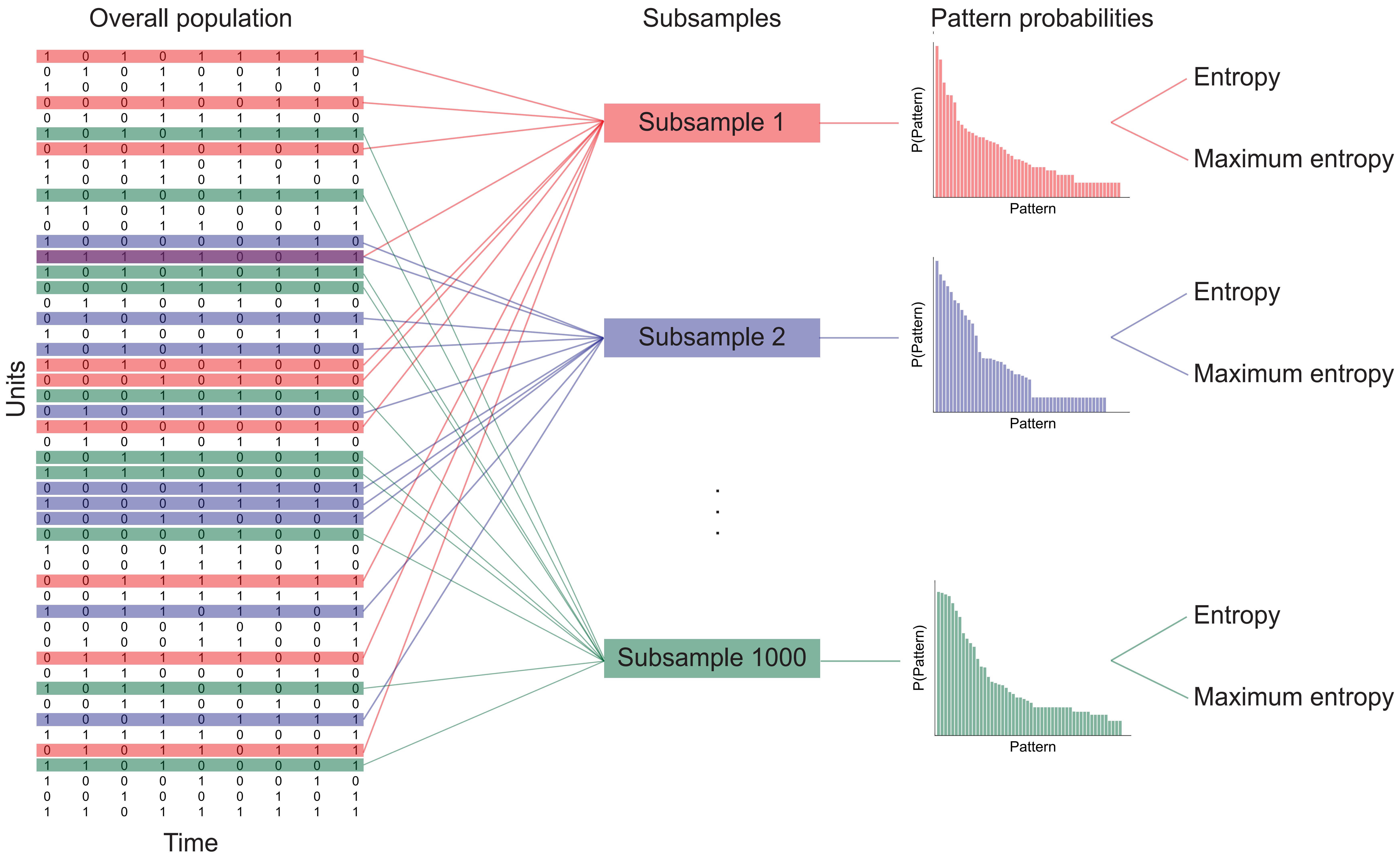

Figure S5 Schematic of subsampling process. Subsets of neurons were repeatedly taken from the overall population to generate 1000 subsamples. For each subsample, pattern probability distributions were generated. In turn, the pattern probability distributions were used to estimate entropy and generate maximum entropy models.

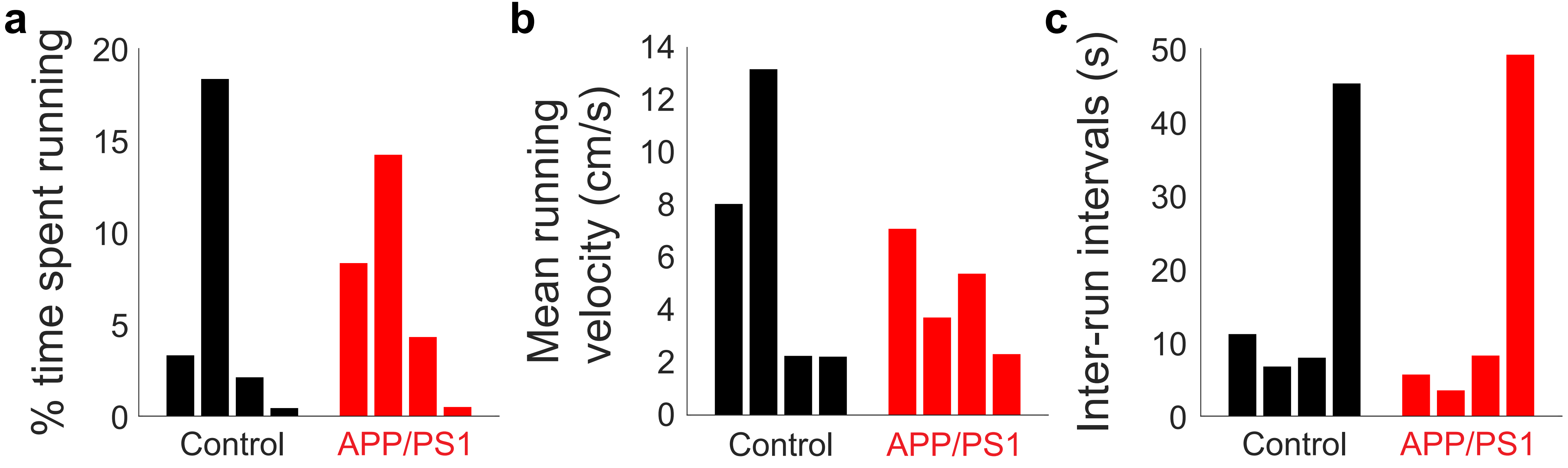

Figure S6 Running statistics for individual animals. Black bars denote control mice and red bars denote APP/PS1 mice. (**a**) Proportion of time spent running, (**b**) mean running velocity, or (**c**) mean IRI length between the two groups.

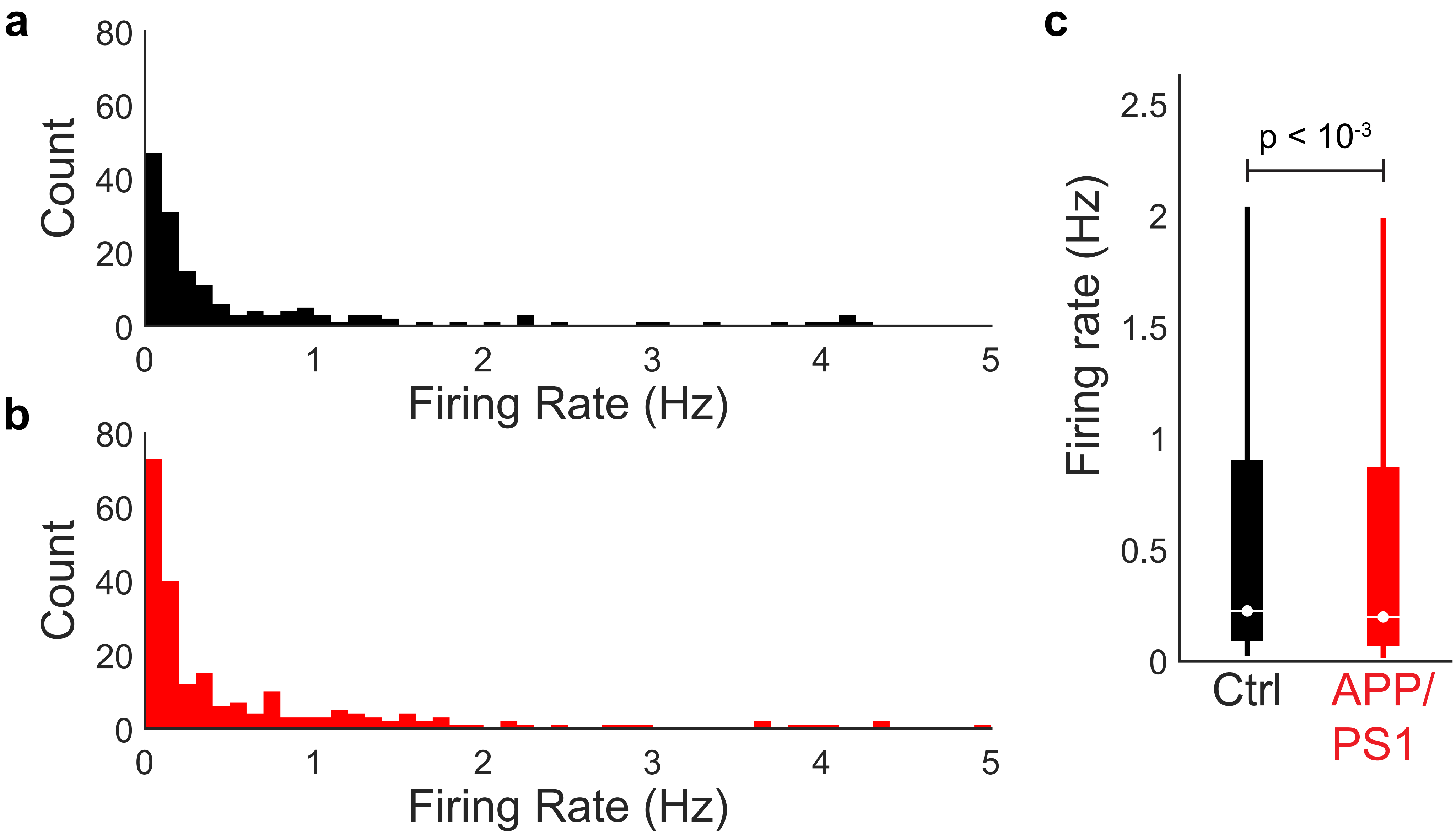

Figure S7 Mean firing rates. (**a**,**b**) Histogram of firing rates from all units in (**a**) control animals and (**b**) APP/PS1 animals. The distribution was not bimodal, as determined by Hartigan's dip test (p = 0.74 for the control distribution and p > 0.99 for the APP/PS1 distribution). (**c**) Box plot of mean firing rates. The APP/PS1 group had significantly lower firing rates than control mice ($p < 10^{-3}$, two-sided Wilcoxon rank-sum test). For both box plots, box center denotes the median, box edges denote the interquartile range (IQR), upper whisker extends to the largest value smaller than 1.5×IQR from upper edge of box, and lower whisker extends to the smallest value larger than 1.5×IQR from lower edge of box.

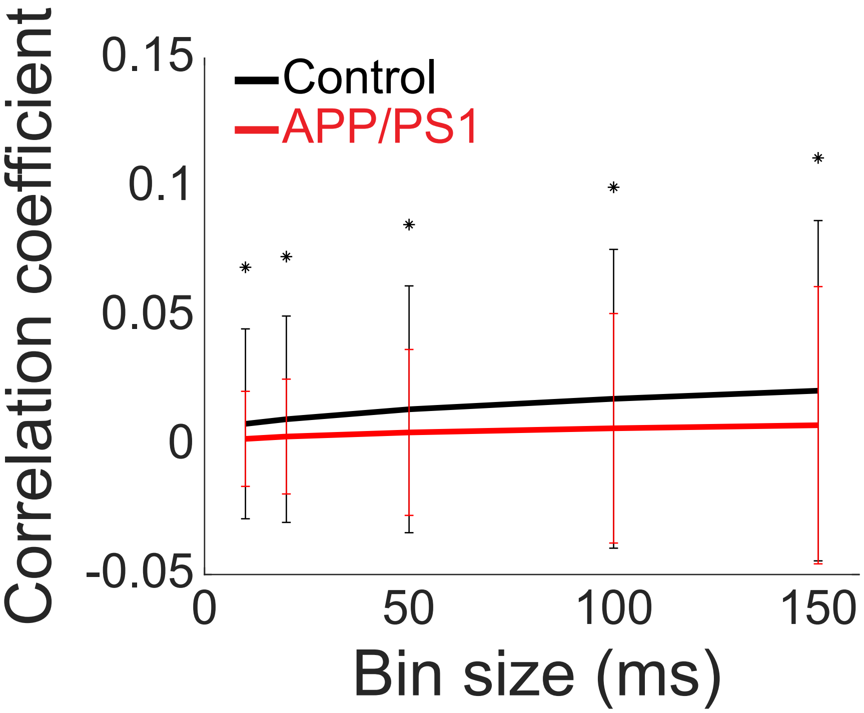

Figure S8 Pearson correlation coefficients of neuronal populations in control and APP/PS1 animals. For all bin sizes examined, correlations were larger for control animals than APP/PS1 animals ($p < 0.05$, Bonferroni corrected, two-sided Wilcoxon rank-sum test). Bold lines denote the mean and error bars denote the standard deviation.

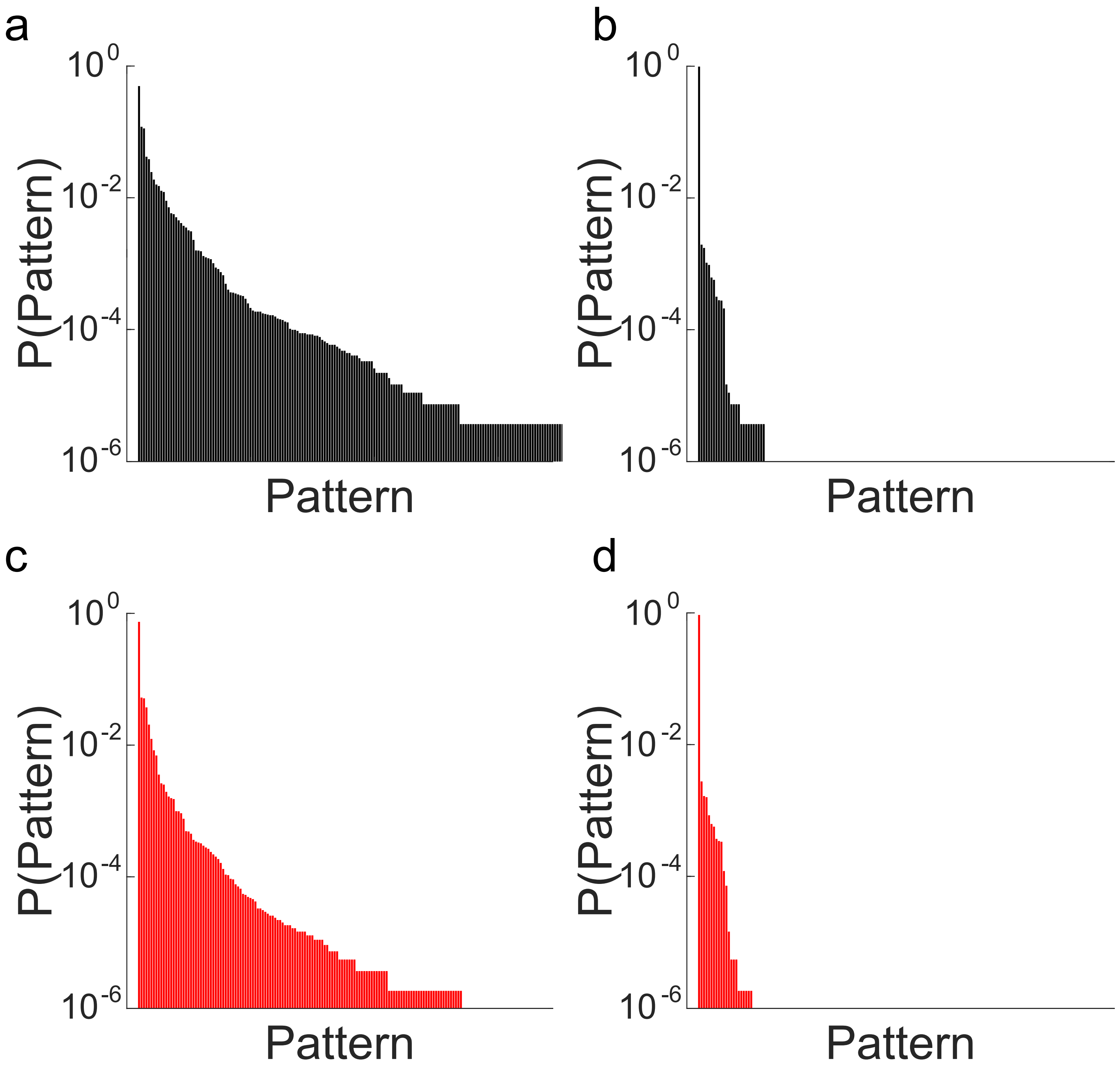

Figure S9 Representative pattern probability distributions from a (**a**,**b**) control and (**c**,**d**) APP/PS1 animal. (**a**,**b**) Example of a high-entropy pattern probability distribution from a single 10-unit subsample in a (**a**) control and a (**c**) APP/PS1 animal. (**c,d**) Example of a low-entropy pattern probability distribution from a single 10-unit subsample in a (**c**) control and (**d**) APP/PS1 animal.

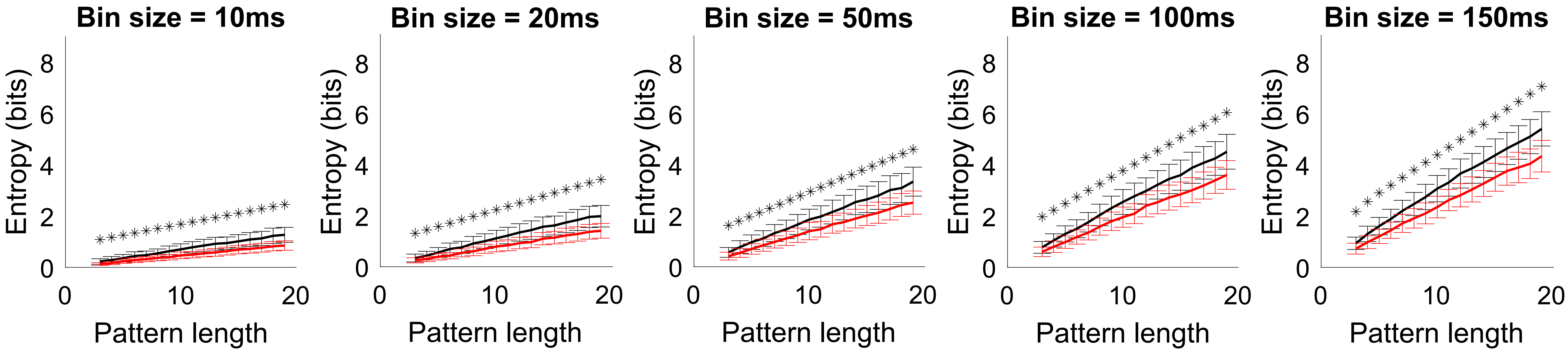

Figure S10 The decreased entropy observed in APP/PS1 animals relative to controls is robust to choice of bin size and pattern length (asterisks denote $p < 0.05$, two-sided Wilcoxon rank-sum test, Bonferroni-corrected). Entropy in both groups increased with pattern length and with bin size. Bold lines denote the mean and error bars denote the standard error of the mean.

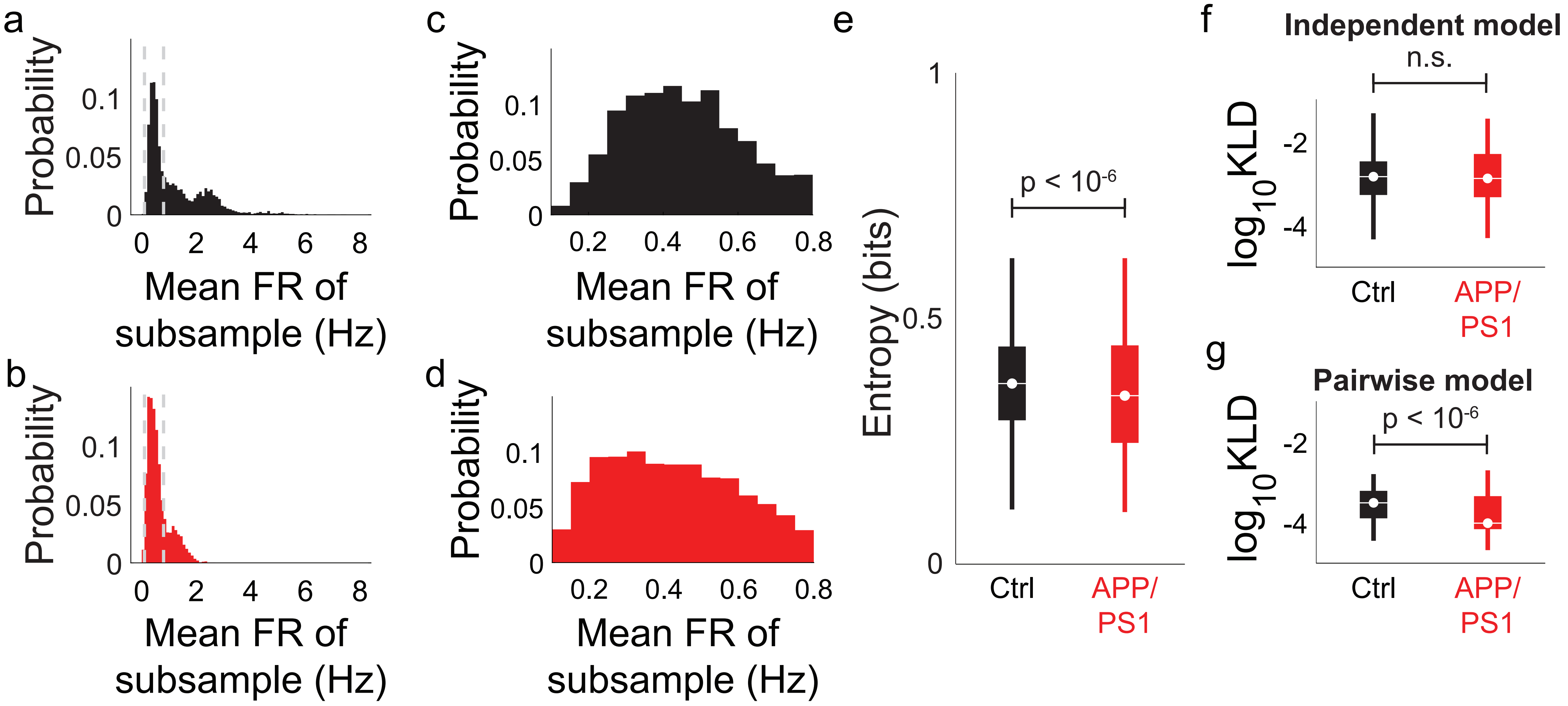

Figure S11 Entropy and maximum entropy model KLD normalized by firing rate (**a**,**b**) Histogram of mean firing rates of 10-unit subsamples from all (**a**) control mice and (**b**) APP/PS1 mice. Dashed grey lines indicate the bounds of an interval (0.1Hz to 0.8Hz) in which a large portion of the distribution of both control and APP/PS1 groups falls. (**c**,**d**) Magnified depiction of the intervals demarcated by dashed grey lines in (**a**) and (**b**). (**e**) Comparison of entropy of only the subsamples that fall within the interval shown in (**c**,**d**). Even after correcting for the differences in mean firing rate between the control and APP/PS1 mice in this way, the APP/PS1 group still had a significantly lower entropy than the control group ($p < 10^{-6}$, two-sided Wilcoxon rank-sum test). (**e**) Comparison of KLD for the independent maximum entropy model for only the subsamples that fall within the interval shown in (**c**,**d**). After correcting for the differences in mean firing rate between the control and APP/PS1 mice in this way, the APP/PS1 group did not have a significantly lower KLD than the control group ($p = 0.15$, two-sided Wilcoxon rank-sum test). (**e**) Comparison of KLD for the pairwise maximum entropy model for only the subsamples that fall within the interval shown in (**c**,**d**). Even after correcting for the differences in mean firing rate between the control and APP/PS1 mice in this way, the APP/PS1 group had a significantly lower KLD than the control group ($p < 10^{-6}$, two-sided Wilcoxon rank-sum test).For all three box plots, box center denotes the median, box edges denote the interquartile range (IQR), upper whisker extends to the largest value smaller than 1.5×IQR from upper edge of box, and lower whisker extends to the smallest value larger than 1.5×IQR from lower edge of box.

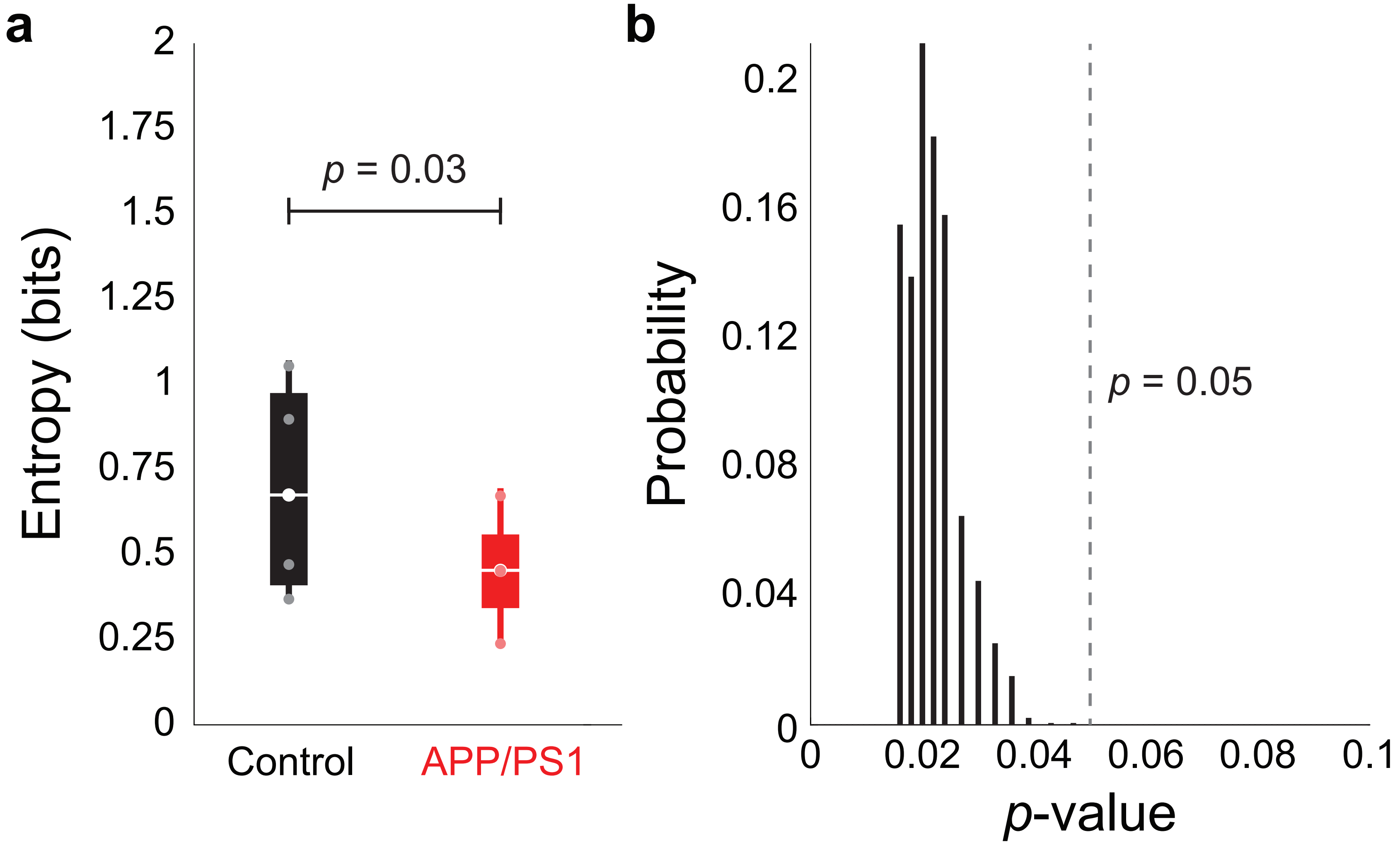

Figure S12 Comparison of entropy values at the single-animal level. (**a**) The entropy distribution of each animal was randomly split into subsets comprised of 250 samples each. The means of these subsets were then compared between control and APP/PS1 animals. This resampling process was performed 5000 times, and a representative example is shown here. At the single-animal level, the entropy of APP/PS1 animals was still less than that of the control animals ($p < 0.05$, two-sided Wilcoxon rank-sum test). Points represent the mean entropy of the subsets from each animal. Box center denotes the median, box edges denote the interquartile range (IQR), upper whisker extends to the largest value smaller than 1.5×IQR from upper edge of box, and lower whisker extends to the smallest value larger than 1.5×IQR from lower edge of box. (**b**) Distribution of *p*-values from all 5000 resamples. All *p*-values are less than 0.05 (dashed line).

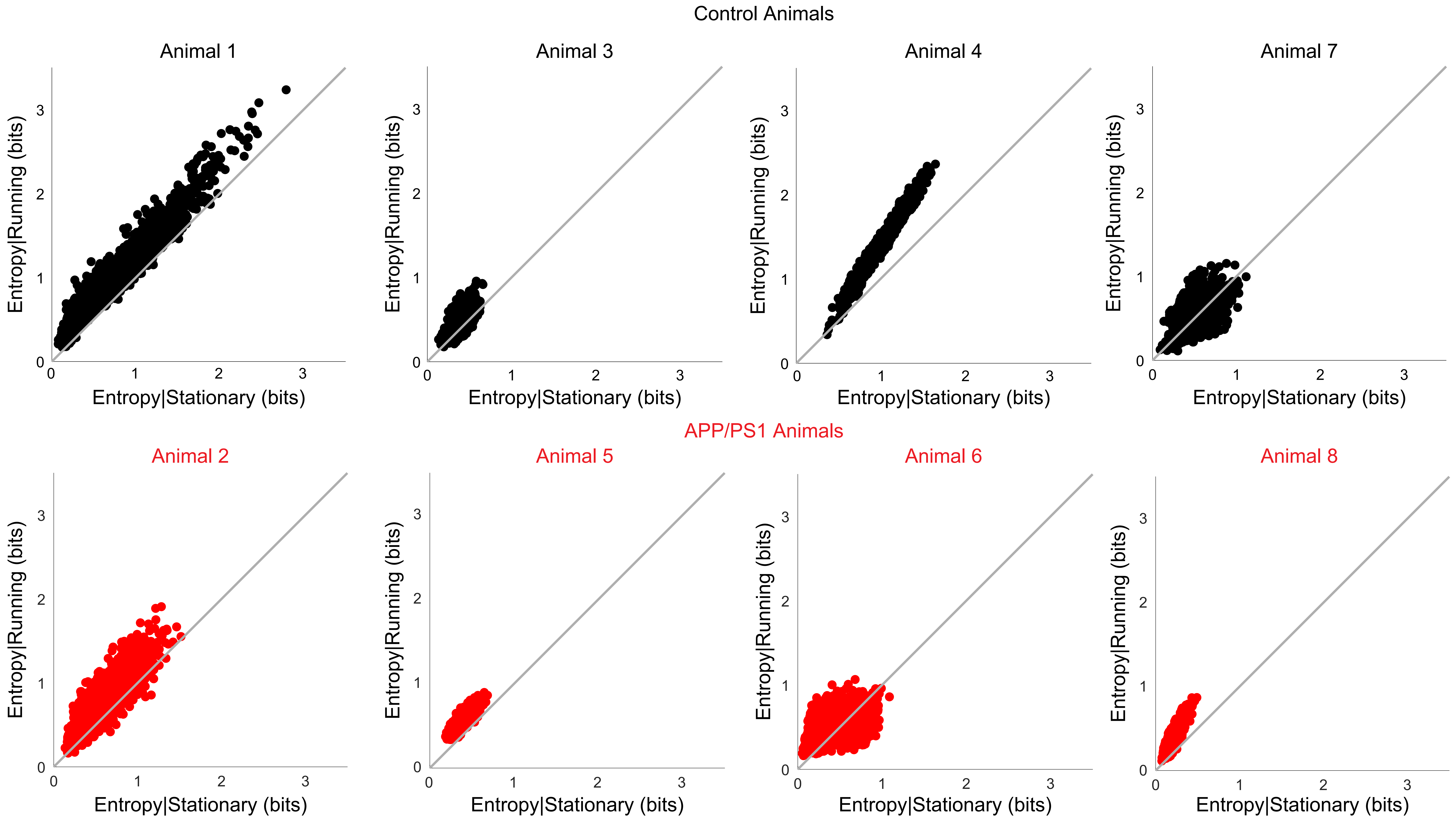

Figure S13 Plots of entropy conditioned on running behavior for each animal. Each point denotes an estimate of conditional entropy based on a single 10-unit subsample of the neuronal population in that animal. Note that for most of the animals, both in the APP/PS1 and the control groups, the points are clustered above the unity line (blue), indicating that running is associated with increases in entropy.

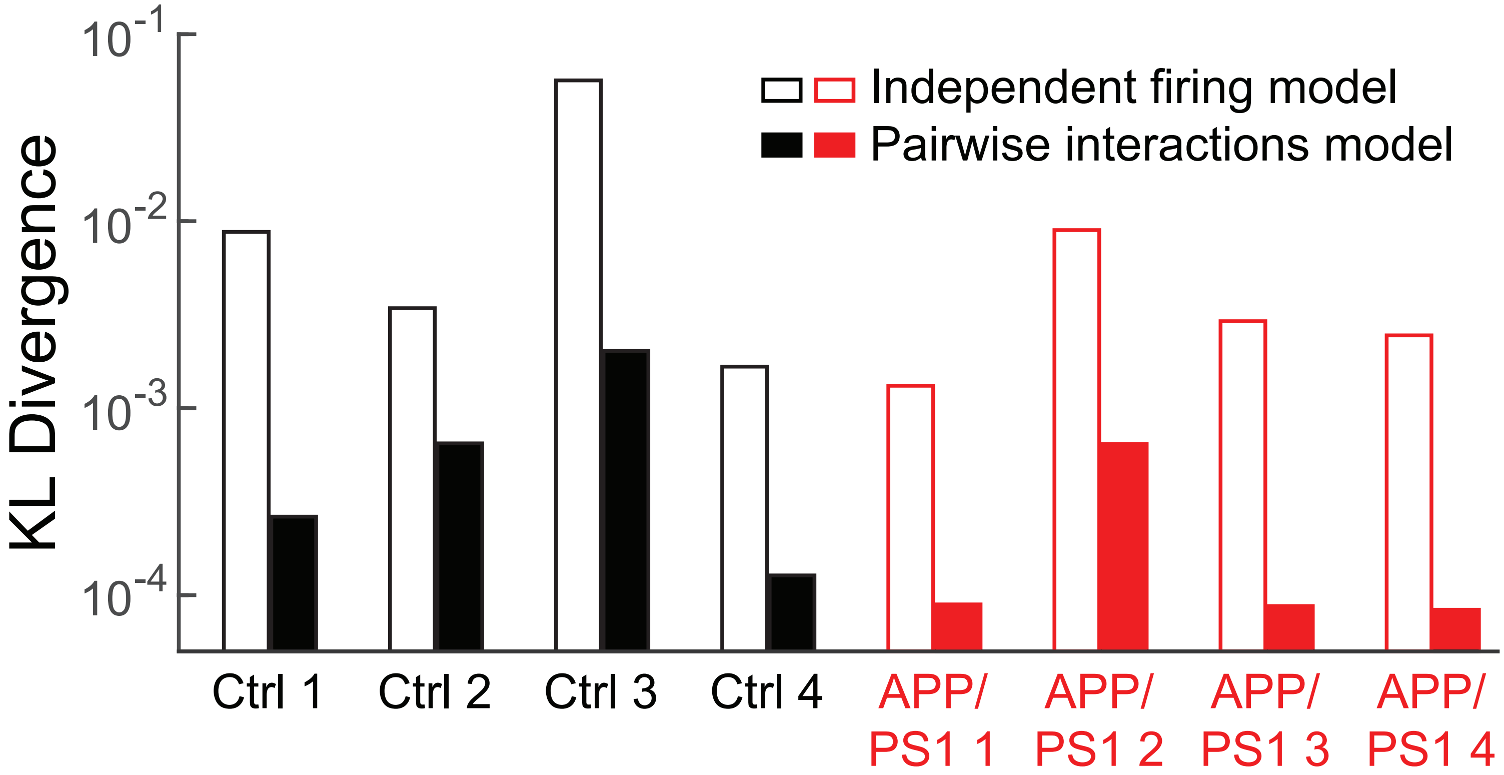

Figure S14 Maximum entropy model fits for each animal. Empty bars show the mean KL divergence of the independent firing model and filled bars show the mean KL divergence of the pairwise interactions model. Control animals are in black and APP/PS1 animals are in red.

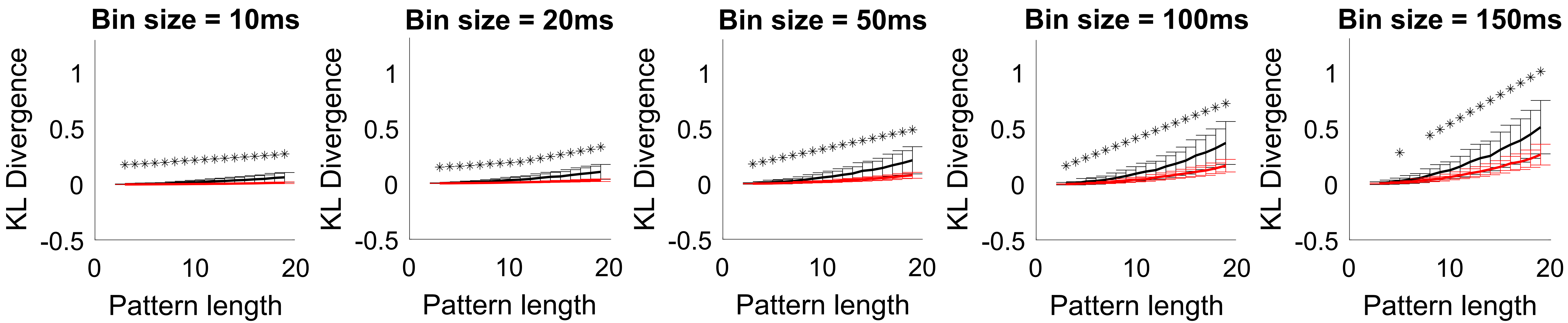

Figure S15 The decreased KL divergence between the empirical and predicted pattern probabilities from the independent firing maximum entropy model observed in APP/PS1 animals relative to controls is robust to choice of bin size and pattern length (asterisks denote $p < 0.05$, two-sided Wilcoxon rank-sum test, Bonferroni-corrected). The KL divergence in both groups increased with pattern length and with bin size. Bold lines denote the mean and error bars denote the standard error of the mean.

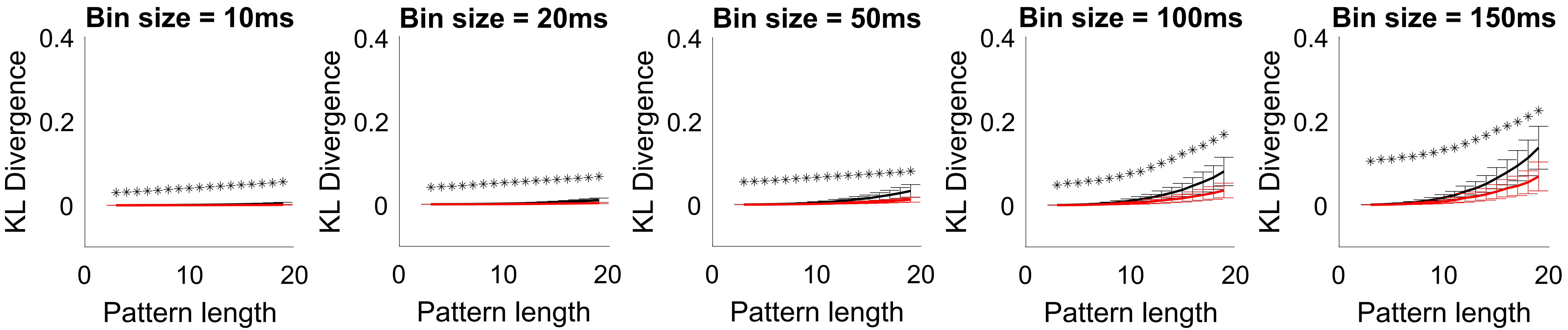

Figure S16 The decreased KL divergence between the empirical and predicted pattern probabilities from the pairwise interactions maximum entropy model observed in APP/PS1 animals relative to controls is robust to choice of bin size and pattern length (asterisks denote $p < 0.05$, two-sided Wilcoxon rank-sum test, Bonferroni-corrected). The KL divergence in both groups increased with pattern length and with bin size. Bold lines denote the mean and error bars denote the standard error of the mean.

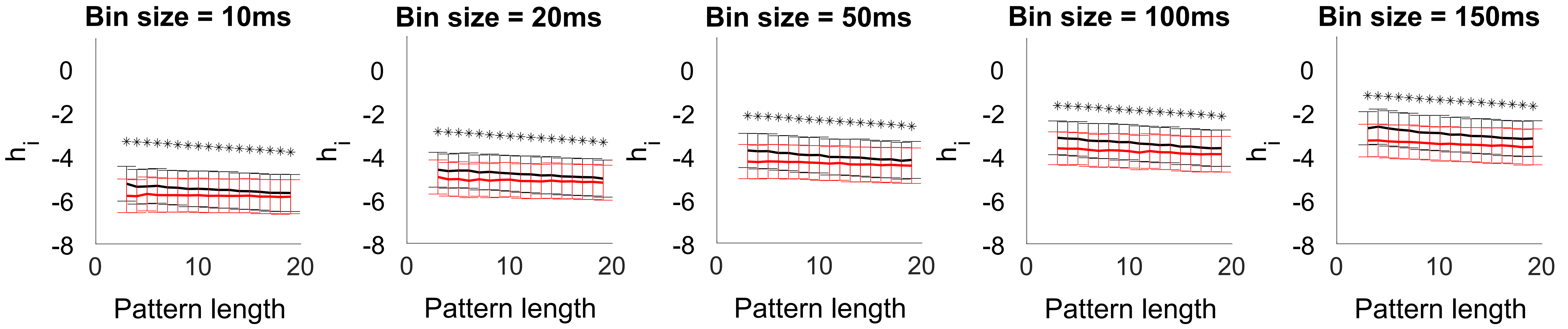

Figure S17 The decreased maximum entropy h_i term observed in APP/PS1 animals relative to controls is robust to choice of bin size and pattern length (asterisks denote $p < 0.05$, two-sided Wilcoxon rank sum test, Bonferroni-corrected). Bold lines denote the mean and error bars denote the standard error of the mean.

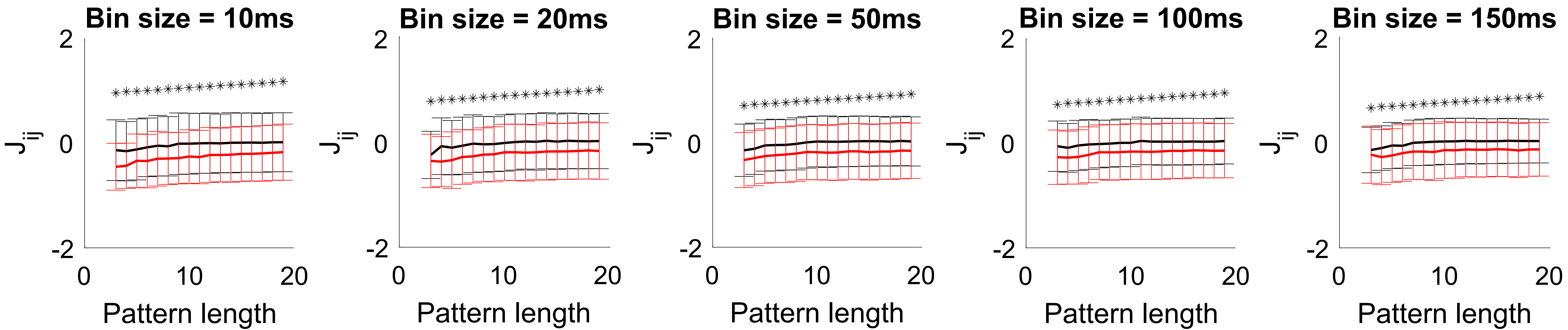

Figure S18 The decreased maximum entropy J_{ij} term observed in APP/PS1 animals relative to controls is robust to choice of bin size and pattern length (asterisks denote $p < 0.05$, two-sided Wilcoxon rank-sum test, Bonferroni-corrected). Bold lines denote the mean and error bars denote the standard error of the mean.

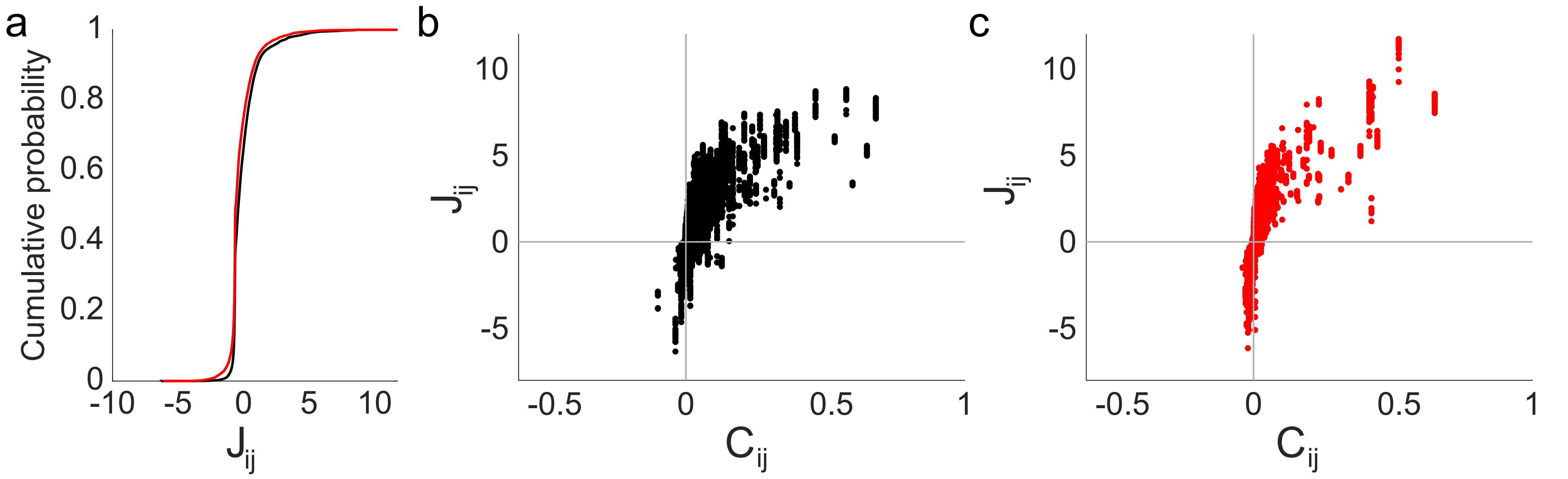

Figure S19 Relationship between the Pearson correlation coefficient C_{ij} and the maximum entropy model pairwise interaction term J_{ij}. (**a**) Cumulative histogram of J_{ij} values for all control (black) and APP/PS1 (red) animals. (**b**,**c**) Scatter plot of C_{ij} and J_{ij} for all control (**b**) and APP/PS1 (**c**) animals.

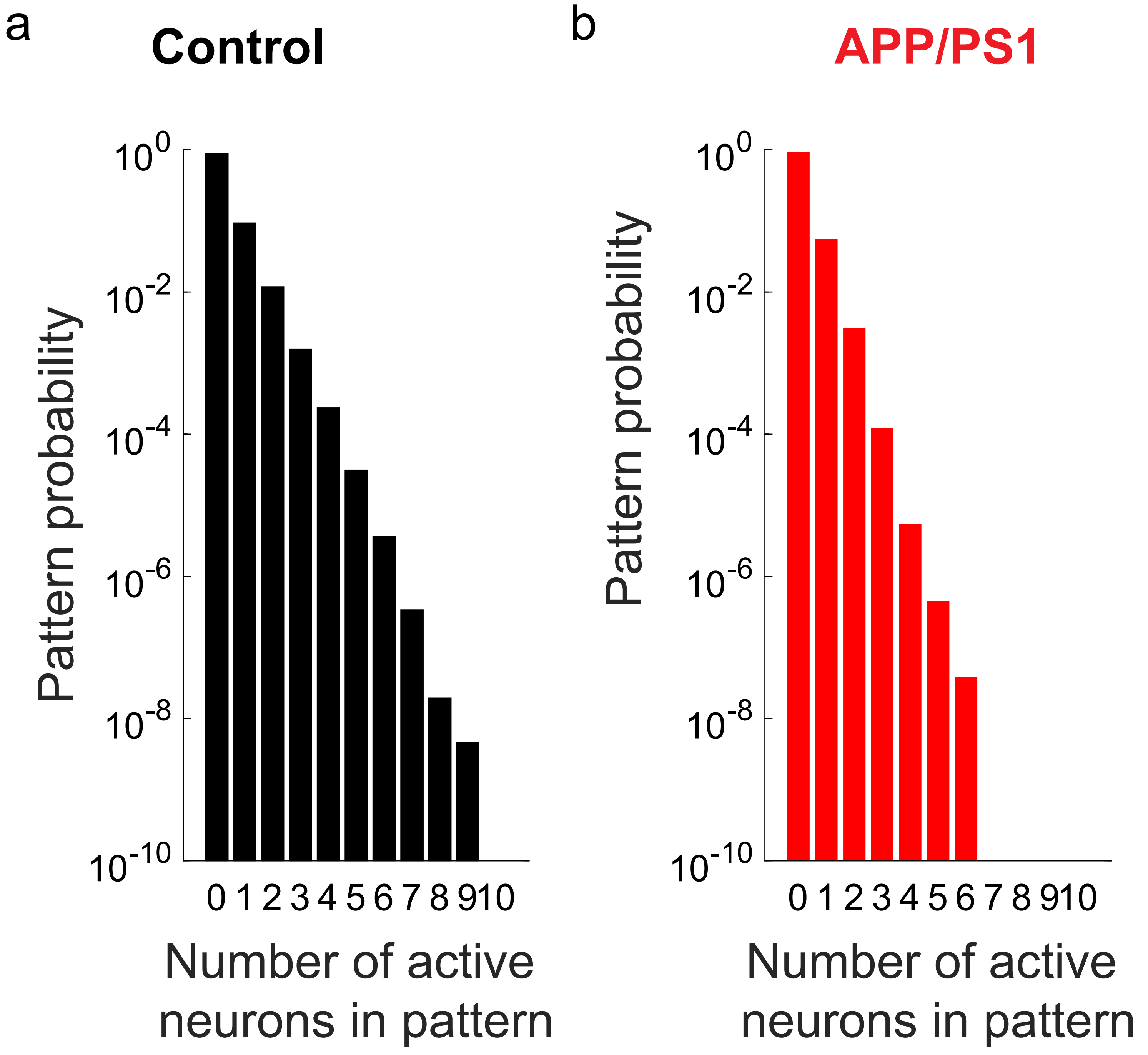

Figure S20 Probability of patterns grouped by number of coactive neurons in (**a**) control and (**b**) APP/PS1 animals.

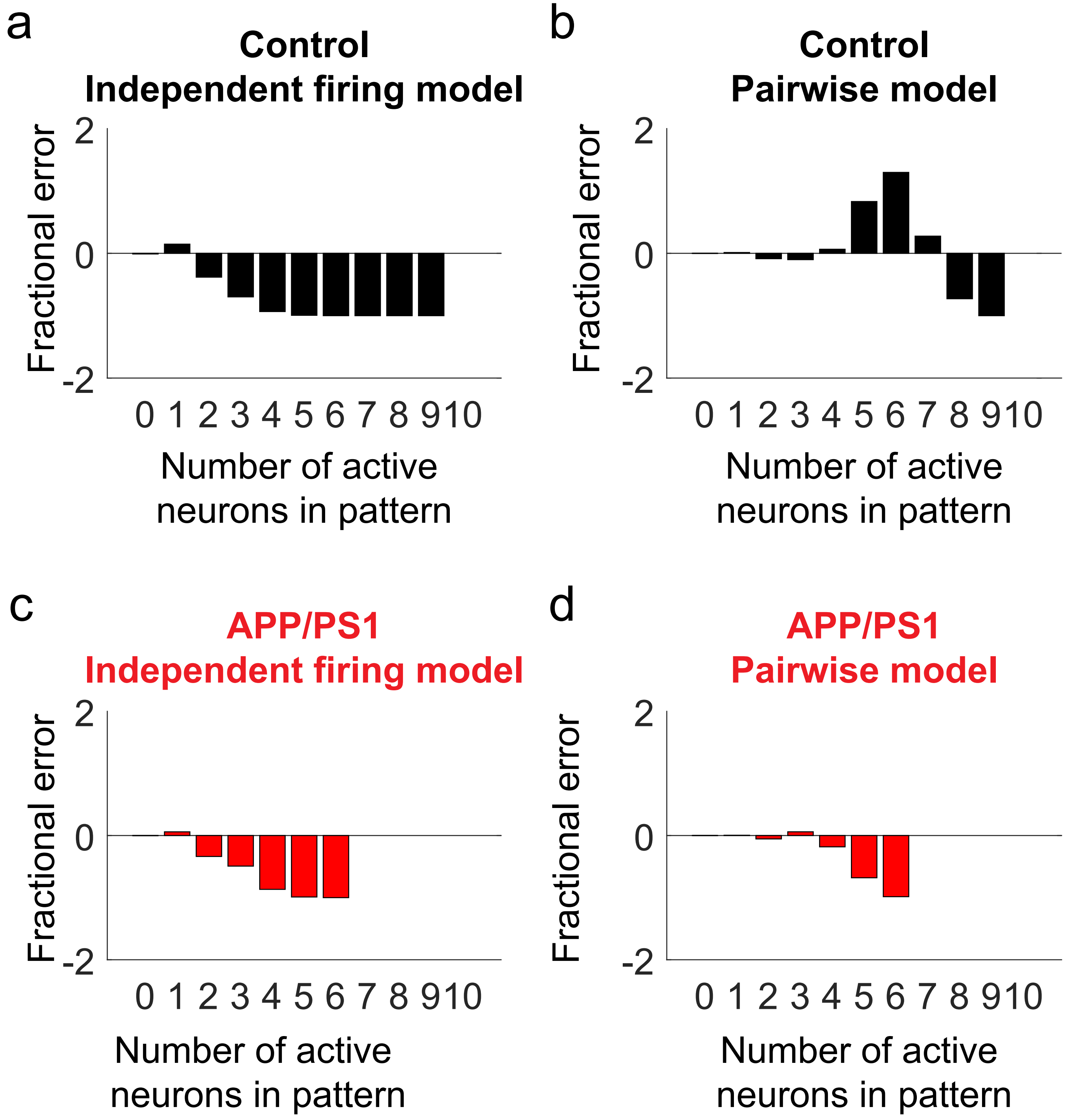

Figure S21 Mean fractional error between predicted and empirical pattern probabilities, grouped by number of coactive neurons in each pattern for (**a**,**b**) control and (**c**,**d**) APP/PS1 animals. (**a**,**c**) Prediction error for the independent firing model. (**b**,**d**) Prediction error for the pairwise interactions model.

Table S1: Summary statistics of all data

Figure	Description	Mean of control group	Standard deviation of control group	Mean of APP/PS1 group	Standard deviation of APP/PS1 group	Effect size (Cohen's d)	Test statistic (U)	p-value	95% CI of difference of control and APP/PS1 medians
3b	% Time running	6.05%	8.28%	6.84%	5.87%	-0.11	6	0.69	(-13.78%,17.85%)
3c	Running velocity	6.42 cm/s	5.27 cm/s	4.63 cm/s	2.07 cm/s	0.45	8	> 0.99	(-4.87 cm/s,10.85 cm/s)
3d	Inter-run interval	17.8 s	18.4 s	16.6 s	21.8 s	0.06	10	0.69	(-42.4 s,41.7 s)
4b	Overall correlation	8.33×10^{-3}	3.68×10^{-2}	2.52×10^{-3}	1.84×10^{-2}	0.23	2.08×10^{7}	$< 10^{-6}$	(8.36×10^{-4},1.17×10^{-3})
4c	Correlation|stationary	8.12×10^{-3}	3.72×10^{-2}	2.55×10^{-3}	1.84×10^{-2}	0.22	2.03×10^{7}	$< 10^{-6}$	(5.91×10^{-4},8.69×10^{-4})
4d	Correlation|running	7.76×10^{-3}	7.66×10^{-2}	2.42×10^{-3}	2.55×10^{-2}	0.12	1.71×10^{7}	$< 10^{-5}$	(-4.91×10^{-4},2.09×10^{-4})
4g	ΔCorrelation with running	-3.62×10^{-4}	7.18×10^{-2}	-1.36×10^{-4}	2.14×10^{-2}	5.38×10^{-2}	1.58×10^{7}	$< 10^{-6}$	(-1.36×10^{-3},9.13×10^{-4})
5b	Overall entropy	0.70 bits	0.42 bits	0.45 bits	0.24 bits	0.72	1.07×10^{7}	$< 10^{-6}$	(0.15 bits,0.18 bits)
5d	Entropy|stationary	0.69 bits	0.42 bits	0.44 bits	0.23 bits	0.73	1.08×10^{7}	$< 10^{-6}$	(0.15 bits,0.18 bits)
5e	Entropy|running	0.89 bits	0.57 bits	0.60 bits	0.25 bits	0.66	9.81×10^{6}	$< 10^{-6}$	(0.12 bits, 0.16 bits)
5h	ΔEntropy with running	0.20 bits	0.21 bits	0.16 bits	0.16 bits	0.23	8.56×10^{6}	$< 10^{-6}$	(1.5×10^{-2} bits,3.3×10^{-2} bits)
6e	$\log_{10}$KLD for independent model	1.76×10^{-2}	3.24×10^{-2}	3.91×10^{-3}	6.26×10^{-3}	0.51	9.98×10^{6}	$< 10^{-6}$	(0.288,0.354)
6e	$\log_{10}$KLD for pairwise model	7.65×10^{-4}	9.46×10^{-4}	2.26×10^{-4}	2.88×10^{-4}	1.13	1.29×10^{7}	$< 10^{-6}$	(0.456,0.500)
6f	h_i	-5.96	1.65	-6.26	1.56	0.19	8.70×10^{8}	$< 10^{-6}$	(0.233,0.281)
6g	J_{ij}	-1.53×10^{-2}	1.15	-0.25	1.04	0.21	1.88×10^{10}	$< 10^{-6}$	(0.101,0.108)

All comparisons were performed using the two-sided Wilcoxon rank-sum test
U is the test statistic of the Wilcoxon rank-sum test
